# Identification of an Evolutionarily Conserved Ankyrin Domain-Containing Protein, Caiap, Which Regulates Inflammasome-Dependent Resistance to Bacterial Infection

**DOI:** 10.3389/fimmu.2017.01375

**Published:** 2017-10-19

**Authors:** Sylwia D. Tyrkalska, Sergio Candel, Ana B. Pérez-Oliva, Ana Valera, Francisca Alcaraz-Pérez, Diana García-Moreno, María L. Cayuela, Victoriano Mulero

**Affiliations:** ^1^Facultad de Biología, Departamento de Biología Celular e Histología, Universidad de Murcia, IMIB-Arrixaca, Murcia, Spain; ^2^Grupo de Telomerasa, Envejecimiento y Cáncer, CIBERehd, Hospital Clínico Universitario Virgen de la Arrixaca, IMIB-Arrixaca, Murcia, Spain

**Keywords:** ankyrin repeats, inflammasome, macrophages, bacterial infection, flagellin

## Abstract

Many proteins contain tandemly repeated modules of several amino acids, which act as the building blocks that form the underlying architecture of a specific protein-binding interface. Among these motifs and one of the most frequently observed is ankyrin repeats (ANK), which consist of 33 amino acid residues that are highly conserved. ANK domains span a wide range of functions, including protein–protein interactions, such as the recruitment of substrate to the catalytic domain of an enzyme, or the assembly of stable multiprotein complexes. Here, we report the identification of an evolutionarily conserved protein, that we term Caiap (from *C*ARD- and *A*NK-containing Inflammasome *A*daptor *P*rotein), which has an N-terminal CARD domain and 16 C-terminal ANK domains and is required for the inflammasome-dependent resistance to *Salmonella* Typhimurium in zebrafish. Intriguingly, Caiap is highly conserved from cartilaginous fish to marsupials but is absent in placental mammals. Mechanistically, Caiap acts downstream flagellin and interacts with catalytic active Caspa, the functional homolog of mammalian caspase-1, through its ANK domain, while its CARD domain promotes its self-oligomerization. Our results therefore point to ANK domain-containing proteins as key inflammasome adaptors required for the stabilization of active caspase-1 in functionally stable, high molecular weight complexes.

## Introduction

The innate immune system detects the presence of microbes and initiates mechanisms to eliminate potentially infectious threats. Microbial detection is achieved through germline-encoded pattern-recognition receptors (PRRs) that survey both the extracellular and intracellular spaces for pathogen-associated molecular patterns (PAMPs) (1). NOD-like receptors (NLRs) are major PRRs responsible for intracellular defense, which mediate caspase-1 processing and, thereby, the activation of pro-inflammatory cytokines, such as interleukin-1β (IL-1β) and IL-18, and the induction of a special program of cell death called pyroptosis (2). The diversity of effector domains (e.g., PYD or CARD) allows the NLRs to interact with a wide variety of binding partners, leading to the activation of multiple signaling pathways and to their oligomerization into multiprotein signaling platforms called inflammasomes (3, 4). Most NLRs have the ability to recruit the adaptor protein ASC, which possesses C-terminal CARD and N-terminal PYD domains. ASC has been shown to form multiprotein complexes with NLRs and caspase-1 *via* PYD–PYD and CARD–CARD homotypic interactions, respectively (5–7).

Many proteins contain repeating amino acid sequences, which act as the building blocks that form the underlying architecture of specific protein-binding interface. Among these amino acid motifs, ankyrin repeats (ANK), consisting of 33 amino acid residues that are highly conserved among many representatives of the plant, animal, and protozoa kingdoms (8, 9), are one of the most frequently observed. ANK was first discovered in the yeast cell cycle regulator Swi6/Cdc10 and the *Drosophila* signaling protein Notch (10), and owes its name to the cytoskeletal protein ankyrin, which contains 24 copies of this repeat (11). Although most proteins with ANK present 6 of these repeats, that number can vary from 2 to 34. Domains containing ANK span a wide range of functions including protein–protein interactions, such as the recruitment of a substrate to the catalytic domain of an enzyme or the assembly of stable multiprotein complexes (12, 13).

Although several key components of the inflammasome have already been characterized in mammals, little is known about the proteins that form part of the inflammasome in other vertebrate groups. Three distinct NLR subfamilies were found when mining genome databases of various non-mammalian vertebrates; the first subfamily (NLR-A) resembles mammalian NODs, the second (NLR-B) resembles mammalian NLRPs, and the third (NLR-C) appears to be unique to ray-finned fish (class Actinopterygii) (14). In addition, while a homolog of ASC was identified in all the non-mammalian species examined, orthologs of caspase-1 seem to be restricted to the superorders Protacanthopterygii (trout and salmon) and Acanthopterygii (seabream, seabass, and medaka) of ray-finned fish (15–17), while most primitive Ostariophysi (catfishes and zebrafish) do not have caspase-1 orthologs. However, a functional homolog of mammalian caspase-1 has been reported in the zebrafish, caspase a (Caspa), which harbors N-terminal PYD and C-terminal CARD domains (18–20).

It has recently been shown that the activation of different inflammasomes is fine-tuned by several proteins, including the interferon-induced guanylate-binding proteins (19, 21–23). Therefore, many pieces are still necessary to solve this puzzle and to elucidate which proteins are involved in inflammasome activation. Here, we report the identification of an evolutionarily conserved N-terminal CARD and C-terminal ANK domains, termed Caiap from *C*ARD- and *A*NK-containing *I*nflammasome *A*daptor *P*rotein, which is required for the inflammasome-dependent resistance to *Salmonella enterica* serovar Typhimurium (ST) *in vivo*.

## Materials and Methods

### Animals

Zebrafish (*Danio rerio* H.) were obtained from the Zebrafish International Resource Center and mated, staged, raised, and processed as described (24). The lines *roy^a9/a9^; nacre^w2/w2^* (*casper*) (25), *Tg(mpx:eGFP)^*i114*^* (26), *Tg(mpeg1:eGFP)^*gl22*^* and *Tg(mpeg1:GAL4)^*gl25*^* (27) have been previously described.

### Sequence Analysis of Caiap in Different Species

Zebrafish Caiap was identified by searching the CARD protein family (PF00619) in the PFAM database.^1^ Zebrafish full-length Caiap sequence was then compared with other known Caiap sequences, obtained from The Universal Protein Resource (UniProt) database,^2^ and with the newly identified variants by multiple sequence alignment carried out with the ClustalX version 2.1 program (28). The molecular weights were estimated using the Protein Molecular Weight tool from The Sequence Manipulation Suite.^3^ The domains of the proteins deduced from the nucleotide sequences were determined using the Simple Modular Architecture Research Tool (SMART), from the European Molecular Biology Laboratory (EMBL) website^4^ (29, 30). Finally, three-dimensional structure predictions were performed using The IntFOLD Integrated Protein Structure and Function Prediction Server^5^ (31) and visualized with The PyMOL Molecular Graphics System, Version 1.8 Schrödinger, LLC.^6^ The ModFOLD Quality Assessment Server (Version 4.0) was used to check the accuracy of the models (32).

### DNA Constructs

The genes encoding zebrafish Caiap (NM_001025492), CaiapΔCARD (deletion from I12 to Y97), wild-type (WT) Flag-Caspa (NM_131505), catalytic inactive Flag-Caspa mutant (C230A) and WT Asc-eGFP (NM_131495) were synthesized by GenScript Corporation. The *cmv*/*sp6:caiap-mCherry, cmv*/*sp6:caiapΔCARD-mCherry*, and *uas:caiap-mCherry; cmlc2:eGFP* constructs were generated by MultiSite Gateway assemblies using LR Clonase II Plus (Life Technologies) according to standard protocols and using Tol2kit vectors described previously (33). The zebrafish Caspa and Asc-Myc expression constructs were previously described (18).

### Morpholino and RNA/DNA/Protein Injection

Specific morpholinos (Gene Tools) were resuspended in nuclease-free water at 1 mM (Table S1 in Supplementary Material). *In vitro*-transcribed RNA was obtained following the manufacturer’s instructions (mMESSAGE mMACHINE kit, Ambion). Morpholinos and RNA were mixed in microinjection buffer and microinjected into the yolk sac of one-cell-stage embryos using a microinjector (Narishige) (0.5–1 nl per embryo). The same amount of MOs and/or RNA was used in all experimental groups. The efficiency of the MOs was checked by assessing caspase-1 activity.

In some experiments, *Tg(mpeg1:GAL4)^*gl25*^* one-cell stage embryos were injected with a solution containing 100 pg *uas:caiap-mCherry; cmlc2:eGFP* construct and 50 pg Tol2 RNA in microinjection buffer (0.5× Tango buffer and 0.05% phenol red solution). Embryos were sorted at 2 dpf according to the presence or absence of green fluorescence in their heart before being infected (see below).

For crispant experiments, sgRNAs obtained by *in vitro* transcription using the MAXIscript T7 Kit (Ambion) were first checked *in vitro* using 100 ng of an amplicon containing the target sequence, 30 nM sgRNA and 30 nM EnGen^®^ Cas9 NLS from *Streptococcus pyogenes* (New England Biolabs). Injection mixes were then prepared with 500 ng/µl Cas9 and 100 ng/µl control (5′-CGTTAATCGCGTATAATACG-3′) or *caiap* (5′-GGGCCACACCGCTGTTGCTG-3′) sgRNA in 300 mM KCl buffer, incubated for 5 min at 37°C and used directly without further storage (34).

### Infection Assays

For most infection experiments, ST 12023 (wild type) and the isogenic derivative mutants for SPI-1/SPI-2 (prgH020:Tn5lacZY ssaV:aphT) (kindly provided by Prof. D. Holden) were used. For some experiments, the ST strains used were: 14028s (wild type) and its isogenic derivatives fliC/fljB mutant and FliCON, which persistently expresses the flagellin protein FliC (35, 36) (kindly provided by Dr. E.A. Miao). Overnight cultures in Luria-Bertani medium (LB) were diluted 1/5 in LB with 0.3 M NaCl, incubated at 37°C until 1.5 optical density at 600 nm was reached, and finally diluted in sterile PBS. Larvae of 2 dpf were anesthetized in embryo medium with 0.16 mg/ml tricaine and 10 or 50 bacteria were injected into the yolk sac (survival curves and caspase-1 activity, respectively) or 100 into the otic vesicle (WISH). Larvae were allowed to recover in egg water at 28–29°C, and monitored for clinical signs of disease or mortality over 5 days. At least three independent experiments were performed with a total number of 300 larvae.

### Tail Fin Wounding

Tail fin amputation was performed at 3 dpf as previously described (37) in casper larvae.

### Caspase-1 Activity Assay

The caspase-1 activity was determined with the fluorometric substrate Z-YVAD-AFC (caspase-1 substrate VI, Calbiochem) as described previously (15, 16, 19). In brief, 25–35 larvae were lysed in hypotonic cell lysis buffer [25 mM 4-(2-hydroxyethyl)piperazine-1-ethanesulfonic acid (HEPES), 5 mM ethylene glycol-bis(2-aminoethylether)-*N*,*N*,*N*′,*N*′-tetraacetic acid (EGTA), 5 mM dithiothreitol (DTT), 1:20 protease inhibitor cocktail (Sigma-Aldrich), pH 7.5] on ice for 10 min. For each reaction, 80 µg protein were incubated for 90 min at 23°C with 50 µM *Z*-YVAD-AFC and 50 µl of reaction buffer [0.2% 3-[(3-cholamidopropyl)dimethylammonio]-1-propanesulfonate (CHAPS), 0.2 M HEPES, 20% sucrose, 29 mM DTT, pH 7.5]. After the incubation, the fluorescence of the AFC released from the Z-YVAD-AFC substrate was measured with a FLUOstart spectrofluorometer (BGM, LabTechnologies) at an excitation wavelength of 405 nm and an emission wavelength of 492 nm. One representative caspase-1 activity assay out of the three carried out is shown accompanying each survival assay.

### Cell Sorting

Approximately 300–500 non-infected and infected larvae from the lines *Tg(mpx:eGFP)^*i114*^* and *Tg(mpeg1:eGFP)^*gl22*^* were anesthetized in tricaine at 24 hpi, minced with a razor blade, incubated at 28°C for 30 min with 0.077 mg/ml Liberase (Roche). The resulting cell suspension was passed through a 40-µm cell strainer. Cell sorting was performed on a FACSCalibur (BD Biosciences) and a SH800Z (Sony).

### Analysis of Gene Expression

Total RNA was extracted from whole embryos/larvae, larval heads, or sorted cells with TRIzol reagent (Invitrogen) following the manufacturer’s instructions and treated with DNase I, amplification grade (1 U/μg RNA; Invitrogen). SuperScript III RNase H^−^ Reverse Transcriptase (Invitrogen) was used to synthesize first-strand cDNA with oligo(dT)18 primer from 1 µg of total RNA at 50°C for 50 min. Real-time PCR was performed with an ABI PRISM 7500 instrument (Applied Biosystems) using SYBR Green PCR Core Reagents (Applied Biosystems). Reaction mixtures were incubated for 10 min at 95°C, followed by 40 cycles of 15 s at 95°C, 1 min at 60°C, and finally 15 s at 95°C, 1 min 60°C, and 15 s at 95°C. For each mRNA, gene expression was normalized to the ribosomal protein S11 (*rps11)* content in each sample using the Pfaffl method (38). Non-infected samples were used as calibrator. The primers used are shown in Table S2 in Supplementary Material. In all cases, each PCR was performed with triplicate samples and repeated with at least two independent samples.

### Whole-Mount *In Situ* Hybridization (WISH)

Transparent Casper embryos were used for WISH (39). *caiap* sense and antisense RNA probes were generated using the DIG RNA Labeling Kit (Roche Applied Science) from linearized plasmids. Embryos were imaged using a Scope.A1 stereomicroscope equipped with a digital camera (AxioCam ICc 3, Zeiss).

### Inflammasome Reconstitution in HEK293T Cells

HEK293T cells (CRL-11268; American Type Culture Collection) were maintained in DMEM:F12 (1:1) supplemented with 10% FCS, 2 mM Glutamax, and 1% penicillin-streptomycin (Life Technologies). Plasmid DNA was prepared using the Mini-Prep procedure (Qiagen). DNA pellets were resuspended in water and further diluted, when required, in PBS. Cells grown on coverslips were transfected with Lipofectamine (Thermofisher), fixed with 4% paraformaldehyde in PBS, incubated 20 min at room temperature with 20 mM glycin, permeabilized with 0.5% NP40 and blocked for 1 h with 2% BSA. Cells were then labeled with anti-FLAG monoclonal (1:7,000) or anti-Myc polyclonal (1:2,000) both from (Sigma-Aldrich), followed by Alexa 488-conjugated secondary antibody (Thermofisher). Samples were mounted using a mounting medium from Dako and examined with a Leica laser scanning confocal microscope AOBS and software (Leica Microsystems). The images were acquired in a 1,024 × 1,024 pixel format in sequential scan mode between frames to avoid cross-talk. The objective used was HCX PL APO CS × 63 and the pinhole value was 1, corresponding to 114.73 µm.

Pull down assays were also performed as described previously (40) with small modifications. HEK293T cells were transfected with the indicated plasmids in each figure, washed twice with PBS, solubilized in lysis buffer (50 mM Tris–HCl, 150 mM NaCl, 1% NP40 and protease inhibitors) during 30 min in agitation and centrifuged (13,000 × *g*, 10 min). Cell lysate (1 mg) was incubated for 2 h at 4°C under gentle agitation with 40 µl of slurry of ANTI-FLAG^®^ M2 or Myc Affinity Gels (Sigma-Aldrich). The immunoprecipitates were washed four times with lysis buffer containing 0.15 M NaCl and then twice with PBS. Finally, the resin was boiled in SDS sample buffer and the bound proteins were resolved on 10 or 15% SDS-PAGE and transferred to nitrocellulose membranes (BioRad) for 50 min at 200 mA. Blots were probed with specific antibodies to FLAG (Sigma-Aldrich), Myc (ThermoFisher), and mCherry (TermoFisher), and then developed with enhanced chemiluminescence reagents (GE Healthcare) according to the manufacturer’s protocol. In some experiments, protein were resolved under non-reducing conditions by omitting SDS and β-mercaptoethanol for the loading buffer.

### Statistical Analysis

Data are shown as mean ± SEM and were analyzed by analysis of variance and a Tukey multiple range test to determine differences among groups. The differences between two samples were analyzed by the Student’s *t*-test. A log rank test with the Bonferroni correction for multiple comparisons was used to calculate the statistical differences in the survival of the different experimental groups. A chi-square test was used to determine differences in the number of specks formed by Caiap in HEK293T cells.

## Results

### Identification and Characterization of Caiap, a Protein Containing a CARD Domain and ANK Repeats, Which Is Highly Conserved from Cartilaginous Fish to Marsupials

**Figure 1:**
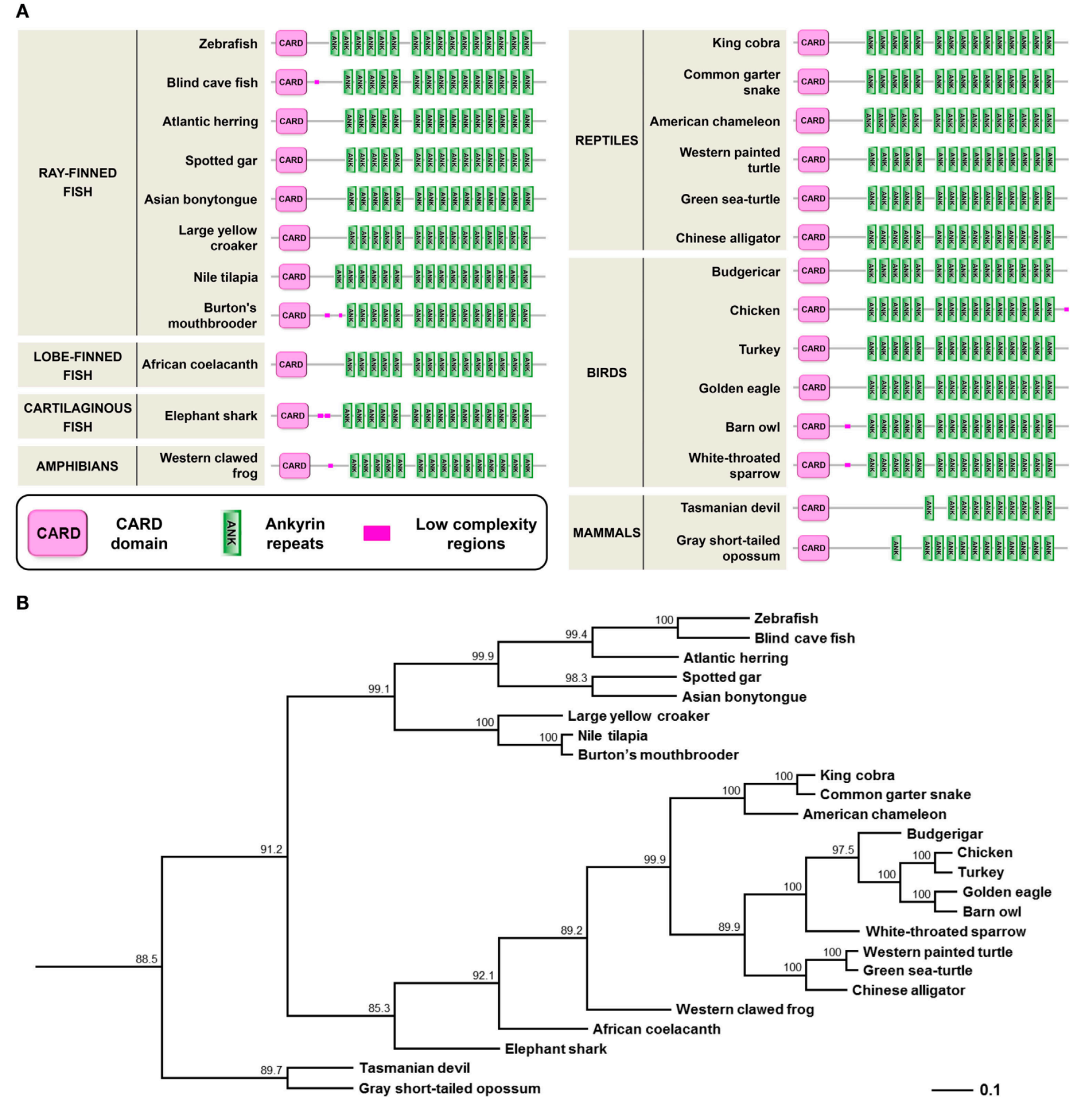
Molecular characteristics and phylogenetic relationships of zebrafish Caiap gene. **(A)** Diagrams showing the domain organization of different fish, amphibians, reptiles, birds, and mammals Caiap. The CARD domains (SMART accession number SM00114) are shown as pink boxes and the ANK repeats (SMART accession number SM00248) are shown as green boxes. **(B)** Phylogenetic tree of vertebrate Caiap polypeptides. The tree was generated by the cluster algorithm using amino acid sequences. Numbers shown are percentages of 100 bootstrap replicates in which the same internal branch was observed. The horizontal lines are drawn proportional to the inferred phylogenetic distances, while the vertical lines have no significance.

A PFAM search to identify proteins harboring CARD domains revealed the presence of Caiap (CARD-ANK Inflammasome Adaptor Protein) in the zebrafish. The *caiap* gene contains two exons and a single open reading frame encoding a putative polypeptide of 744 amino acids (predicted molecular mass of 80.9 kDa) with an N-terminal CARD domain and 16 C-terminal ANK repeats (Figure S1 in Supplementary Material; Figure 1A). Strikingly, uncharacterized orthologs of zebrafish Caiap were found in many organisms, including phylogenetically distant ray-finned fish species, cartilaginous fish (elephant shark), lobe-finned fish (coelacanth), amphibian, reptiles, birds, and marsupials. However, we failed to find a Caiap ortholog in placental mammals, invertebrates, protochordates (amphioxus and sea squirts), jawless fish (lamprey), and lung fish by using homology and synteny searches. Phylogenetic analysis confirmed that the origin of Caiap predated the split of fish and tetrapods more than 450 million years ago, suggesting, therefore, that Caiap was lost in lung fish and placental mammals during evolution (Figure 1B).

**Figure 2:**
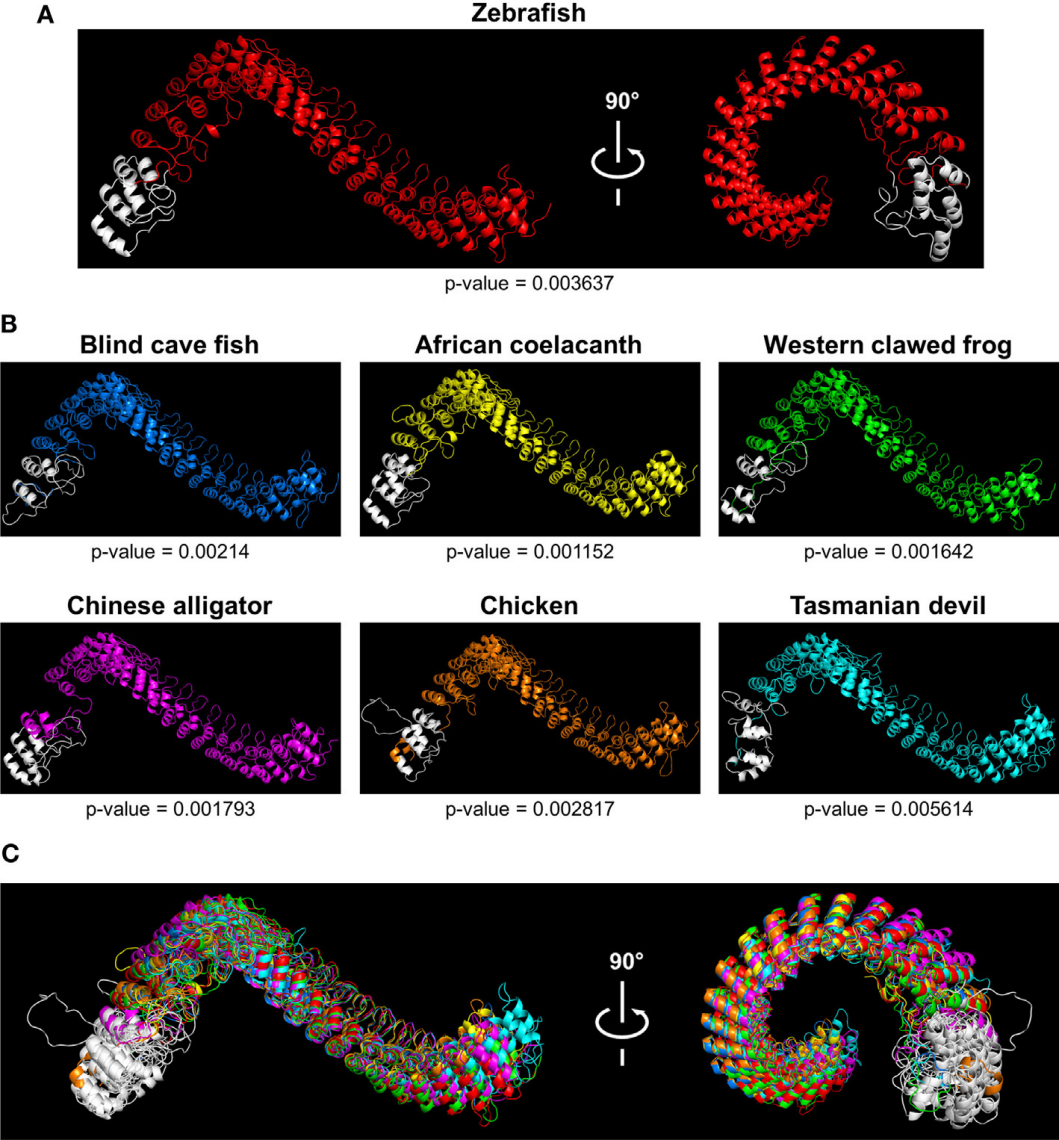
Tridimensional models of Caiap structures in different species. **(A,B)** 3D models showing Caiap proteins from zebrafish (*Danio rerio*) **(A)**, and blind cave fish (*Astyanax fasciatus mexicanus*), western clawed frog (*Xenopus tropicalis*), African coelacanth (*Latimeria chalumnae*), Chinese alligator (*Alligator sinensis*), chicken (*Gallus gallus*), and Tasmanian devil (*Sarcophilus harrisii*) **(B)** with corresponding accuracies. **(C)** The 3D Caiap models from all species shown in **(A,B)** were superimposed. The CARD domains are shown in white.

Caiap was seen to be well conserved across vertebrate species. Thus, zebrafish Caiap showed from 47 to 66% amino acid identity and from 64 to 69% amino acid similarity with Caiap from other ray-finned fish species (Table 1). In addition, a similar degree of conservation was found between zebrafish Caiap and those from other vertebrate groups, with the exception of marsupials (35% identity and 55% amino acid similarity) (Table 1). Multiple alignment of all Caiap identified revealed that the CARD domain and ANK repeats were the best conserved regions of the protein (Figure S1 in Supplementary Material). More interestingly, three-dimensional structural prediction revealed an identical tertiary structure of Caiap in all vertebrate groups (Figures 2A,B). In fact, all the structures perfectly fitted when superimposed (Figure 2C).

**Table 1:** Amino acid identity and similarity between zebrafish Caiap and other vertebrate Caiap sequences.

Species	Identity/similarity (%)
**Ray-finned fish**	
Blind cave fish	66.0/79.4
Atlantic herring	60.2/74.3
Spotted gar	55.3/73.5
Asian bonytongue	52.9/70.4
Large yellow croaker	50.0/66.4
Nile tilapia	48.4/64.8
Burton’s mouthbrooder	47.7/64.1
**Lobe-finned fish**	
African coelacanth	49.9/69.1
**Cartilaginous fish**	
Elephant shark	48.2/65.2
**Reptiles**	
American chameleon	50.1/70.4
Common garter snake	50.0/69.2
Chinese alligator	49.8/70.5
Western painted turtle	49.7/70.1
King cobra	49.6/68.5
Green sea-turtle	48.2/68.9
**Birds**	
Turkey	49.6/67.4
Chicken	49.5/67.8
Budgerigar	49.3/67.8
Golden eagle	48.9/67.3
Barn owl	48.2/67.0
White-throated sparrow	48.0/65.0
**Amphibians**	
Western clawed frog	47.5/68.5
**Mammals**	
Tasmanian devil	35.7/57.2
Gray short-tailed opossum	35.3/55.4

*The accession numbers are XP_685576 for zebrafish (*Danio rerio*), XP_007259539 for blind cave fish (*Astyanax fasciatus mexicanus*), XP_012694853 for atlantic herring (*Clupea harengus*), XP_006635081 for spotted gar (*Lepisosteus oculatus*), KPP59498 for Asian bonytongue (*Scleropages formosus*), KKF28470 for large yellow croaker (*Pseudosciaena crocea*), XP_006002757 for African coelacanth (*Latimeria chalumnae*), XP_005479286 for Nile tilapia (*Oreochromis niloticus*), XP_007901907 for elephant shark (*Callorhinchus milii*), XP_014189326 for Burton’s mouthbrooder (*Haplochromis burtoni*), XP_008107583 for American chameleon (*Anolis carolinensis*), XP_013917140 for common garter snake (*Thamnophis sirtalis*), XP_006025802 for Chinese alligator (*Alligator sinensis*), XP_005284853 for western painted turtle (*Chrysemys picta bellii*), ETE70266 for king cobra (*Ophiophagus hannah*), XP_007059125 for green sea-turtle (*Chelonia mydas*), XP_010714589 for turkey (*Meleagris gallopavo*), XP_004936902 for chicken (*Gallus gallus*), XP_005151355 for budgerigar (*Melopsittacus undulatus*), XP_011570978 for golden eagle (*Aquila chrysaetos canadensis*), XP_009961493 for barn owl (*Tyto alba*), XP_005482422 for white-throated sparrow (*Zonotrichia albicollis*), XP_002931656 for western clawed frog (*Xenopus tropicalis*), XP_003767351 for Tasmanian devil (*Sarcophilus harrisii*), and XP_007480566 for gray short-tailed opossum (*Monodelphis domestica*)*.

### Zebrafish Caiap Is Induced upon Infection

**Figure 3:**
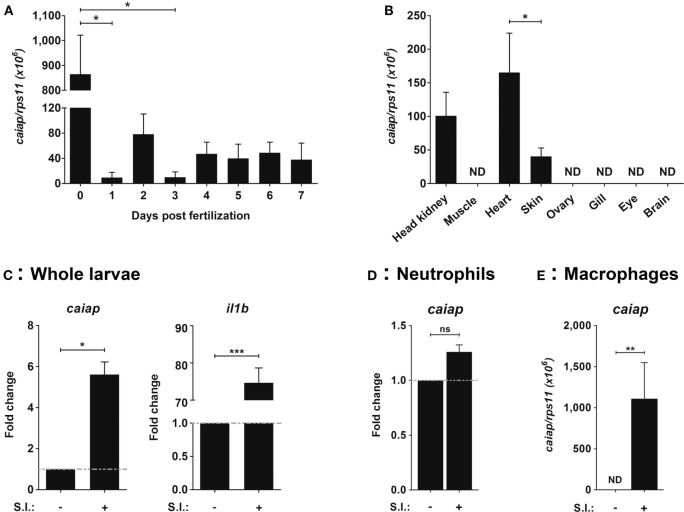
Zebrafish *caiap* is induced in macrophages upon ST infection. The *caiap* **(A–E)** and *il1b* **(C)** mRNA levels were measured by RT-qPCR in wild-type 0–7 dpf whole larvae **(A)**, in head kidney, muscle, heart, skin, ovary, gills, eye, and brain of 12-month-old wild-type adult fish **(B)**, and whole larvae **(C)**, neutrophils **(D)**, and macrophages **(E)** from 3 dpf larvae which were previously infected with ST or not at 2 dpf (n = 3). ns, not significant; *p < 0.05; **p < 0.01; ***p < 0.001. ND, not detected.

The expression profile of zebrafish Caiap was examined using RT-qPCR and WISH. It was found that the mRNA levels of *caiap* transcripts were maternally transferred, since they peaked at fertilization time and then rapidly declined (Figure 3A). In adult fish, *caiap* transcripts were detected in kidney, the hematopoietic organ of adult fish, heart, and skin, but not in muscle, ovary, gills, eye, or brain (Figure 3B). In addition, the mRNA levels of *caiap* were seen to be weakly higher 24 h post-infection (hpi) in the infection site of zebrafish larvae infected with ST (Figure 3C). As expected, the gene encoding pro-inflammatory IL-1β robustly increased in infected larvae (Figure 3C).

**Figure 4:**
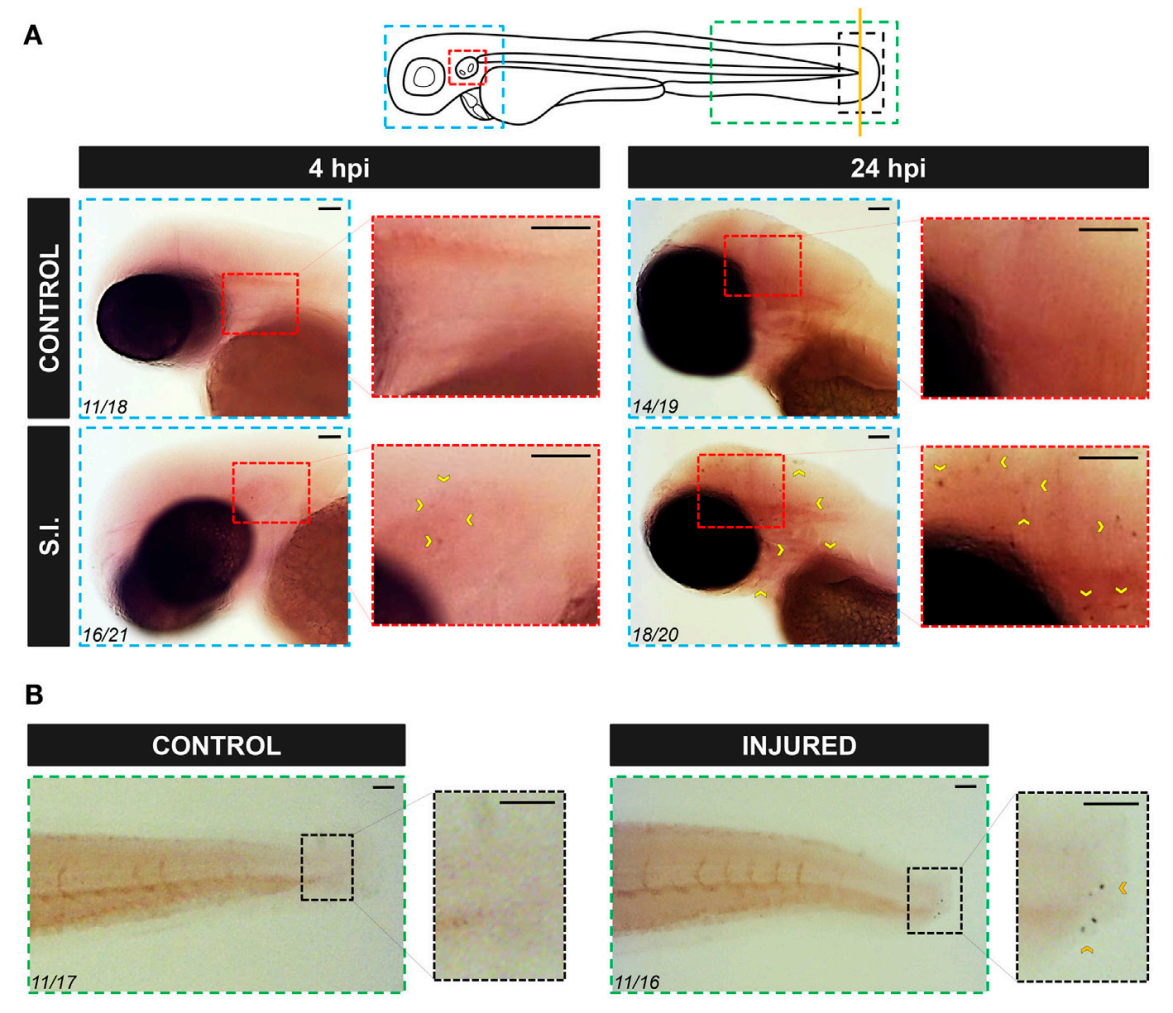
Zebrafish *caiap* is induced in discrete cells at the infection site. Zebrafish 2 dpf larvae were infected with ST in the otic vesicle **(A)** or tail wounded **(B)**. At the indicated times, WISH was performed using antisense probes to the *caiap* gene. Note the presence of *caiap*^+^ cells at both the infection and injured sites (arrowheads). The areas shown are indicated in the larval scheme with boxes of different colors. Numbers in pictures represent the animals with the shown phenotype per total analyzed animals. S.I., ST infection. Scale bar: 0.5 mm.

To further confirm the RT-qPCR results, WISH was performed in infected and wounded larvae. Although no positive cells were observed in non-infected larvae, a few small round *caiap*^+^ positive cells were found at the infection site (the otic vesicle), the number of positive *caiap*^+^ cells increasing from 4 to 24 hpi (Figure 4A). Similarly, a few *caiap*^+^ cells were also observed in the wound 24 h after transection of the tail fin tip (Figure 4B). As expected, no positive cells were observed when using the *caiap* sense probe (Figure S2 in Supplementary Material). This result suggested that both infection and wounding are able to induce the expression of *caiap* in immune cells recruited to the infection and wounding sites, namely, macrophages (41, 42) and neutrophils (19, 37, 43). Therefore, macrophages and neutrophils were sorted from *mpeg1:eGFP* (27) and *mpx:eGFP* (26) transgenic larvae, respectively, upon infection with ST and the transcript levels of *caiap* were analyzed by RT-qPCR in both cells types. The results showed that while *caiap* transcripts drastically increased in macrophages upon ST infection (Figure 3D), they remain unaltered in neutrophils upon infection (Figure 3E).

### Caiap Is Required for the Inflammasome-Dependent Resistance to ST in Zebrafish

**Figure 5:**
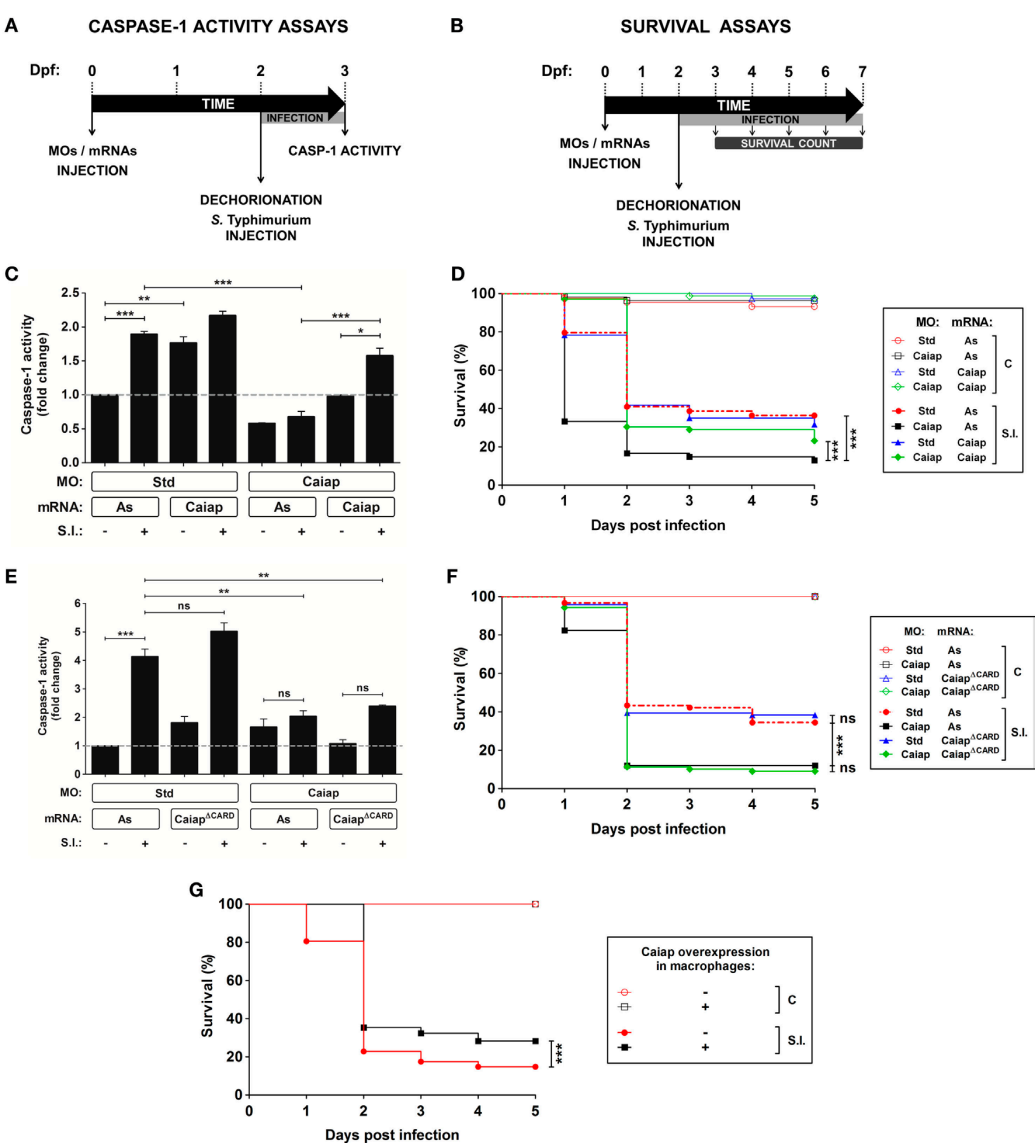
Zebrafish Caiap is required for the inflammasome-dependent resistance to *S*. Typhimurium. **(A)** Scheme showing the experimental procedure used for the caspase-1 activity assays. Zebrafish one-cell embryos were injected with MOs and/or mRNAs, dechorionated and infected at 2 dpf *via* the yolk sac with ST with a MOI of 50, and collected and pooled (25–35 larvae) at 24 hpi to measure caspase-1 activity. **(B)** Scheme showing the experimental procedure used for the survival assays. Zebrafish one-cell embryos were injected with MOs and/or mRNAs, dechorionated and infected at 2 dpf *via* the yolk sac with ST at a MOI of 10, and the number of surviving larvae counted daily during the next 5 days. At least three independent experiments were performed with a total number of 300 specimens/treatment. **(C–F)** Zebrafish one-cell embryos were injected with standard control (Std) **(C–F)** or Caiap MOs **(C–F)**, or with antisense (As) **(C–F)**, Caiap **(C,D)** or CaiapΔCARD **(E,F)** mRNAs, infected at 2 dpf with wild-type ST and the caspase-1 activity **(C,E)** and survival **(D,E,G)** were determined as described in Figures 5A,C, respectively. The sample size for each treatment is 30 for **(C,E)**, and 300 for **(D,F,G)**. S.I., ST infection. **p < 0.01; ***p < 0.001.

To further characterize zebrafish Caiap we used a morpholino (MO)-mediated gene knockdown strategy whereby the MO was able to bind the translation start of the *caiap* mRNA and, therefore, to inhibit its translation (Figure S1A in Supplementary Material). The efficiency of the MO was validated by using a caspase-1 activity assay with a fluorogenic substrate, Z-YVAD-AFC (Figures 5A,C), which has previously been shown to be processed by gilthead seabream caspase-1 (15, 19, 44) and zebrafish Caspa (18). Caiap morphant animals injected with 1 pg/egg MOs showed reduced caspase-1 activity but they developed normally and were viable (Figures 5C,D). However, Caiap-deficient larvae showed increased susceptibility to WT ST compared with their control siblings (Figure 5D) and impaired caspase-1 activity in response to the infection (Figure 5C). Additionally, infection susceptibility and decreased caspase-1 activity were both fully reversed by injection of non-targetable *caiap* mRNA, confirming the specificity of the MO (Figures 5C,D). In contrast, forced expression of CaiapΔCARD failed to rescue the high susceptibility and impaired caspase-1 activation of Caiap-deficient larvae (Figures 5E,F), suggesting that the CARD domain is indispensable for Caiap function. Furthermore, Caiap crispant larvae also showed higher susceptibility to ST infection and reduced caspase-1 activity upon infection than their WT siblings (Figure S3 in Supplementary Material).

**Figure 6:**
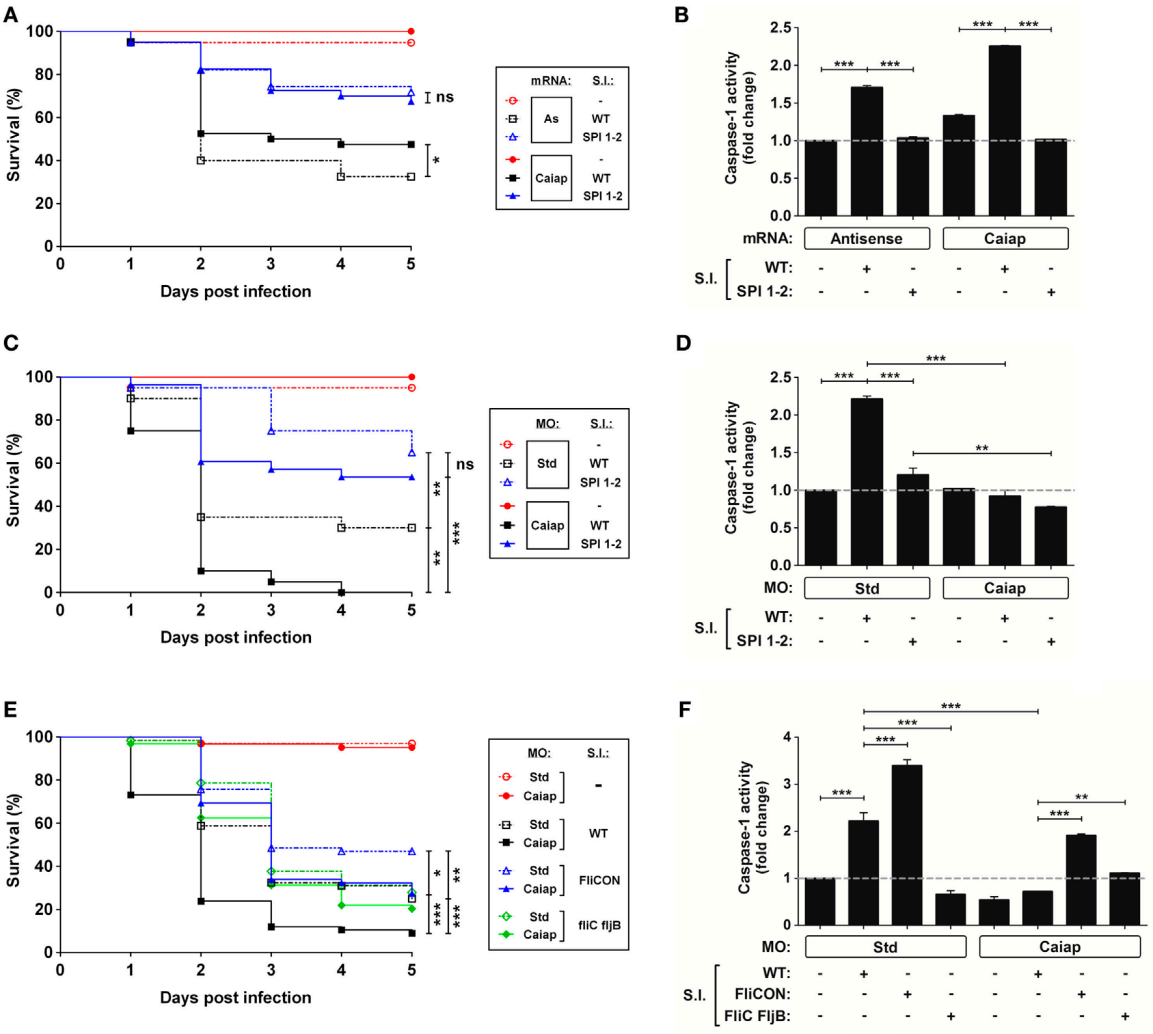
The type 3 secretion system and flagellin of *S*. Typhimurium act upstream Caiap. **(A–F)** Zebrafish one-cell embryos were injected with standard control (Std) or Caiap MOs, or with antisense (As) or Caiap mRNAs, infected at 2 dpf with wild-type (WT) (A-F), the double mutant SPI 1–2 **(A–D)**, FliCON or FliC FljB ST **(E,F)** and the survival **(A,C,E)** and caspase-1 activity **(B,D,F)** were determined as described in Figures 5A,B, respectively. The sample size for each treatment is 300 for **(A,C,E)**, and 30 for **(B,D,F)**. S.I., ST infection; ns, not significant; *p < 0.05; **p < 0.01; ***p < 0.001.

**Figure 7:**
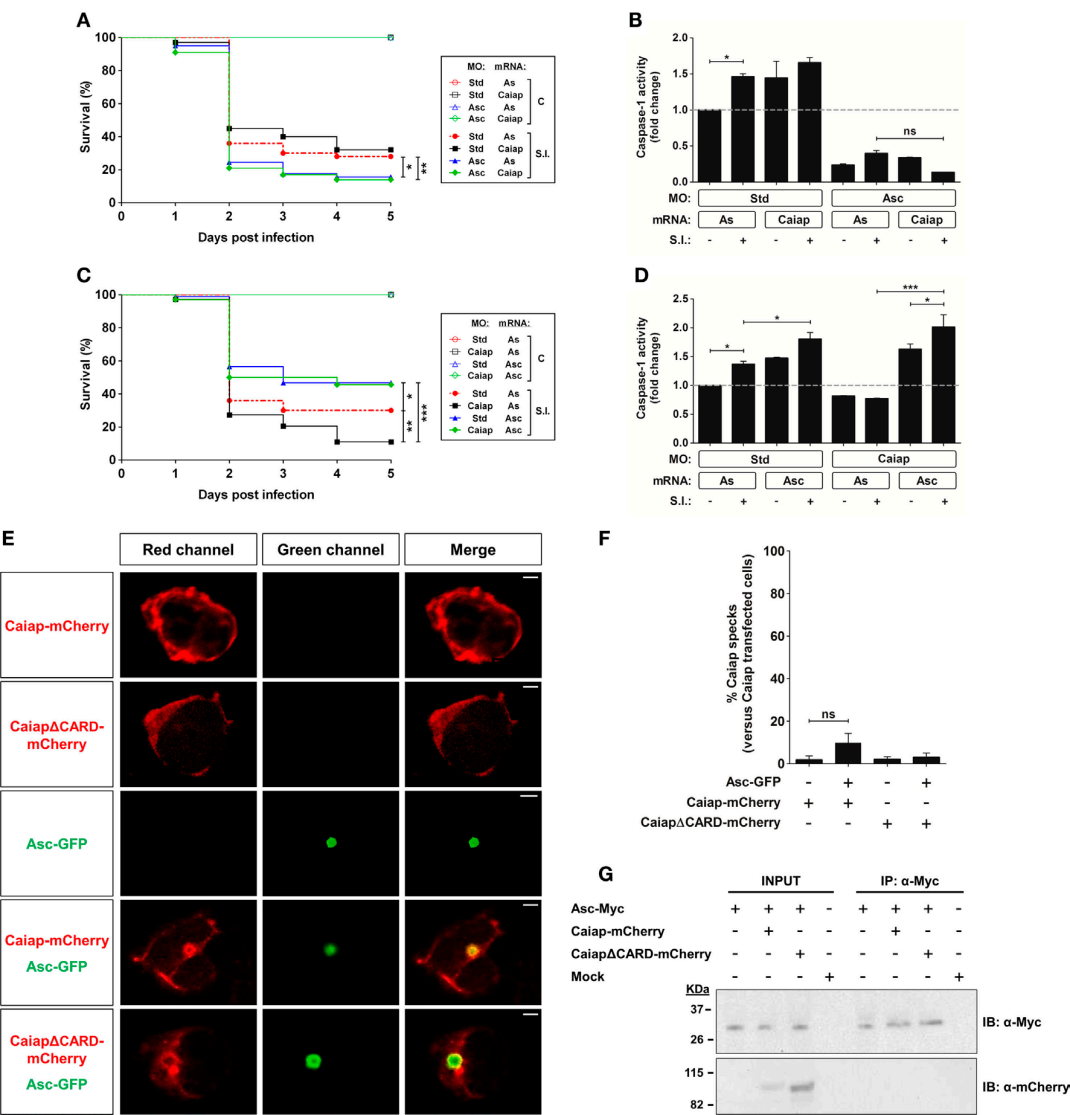
Asc is required for the Caiap-mediated resistance to *S*. Typhimurium. **(A–D)** Zebrafish one-cell embryos were injected with standard control (Std) **(A–D)**, Asc **(A,B)** or Caiap **(C,D)** MOs or in combination with antisense (As) **(A–D)**, Caiap **(A,B)** or Asc **(C,D)** mRNAs, infected at 2 dpf and survival **(A,C)** and caspase-1 activity **(B,D)** determined as described in Figures 5A,B, respectively. S.I., ST infection. **(E–G)** HEK293T cells were transfected with zebrafish Caiap-mCherry or CaiapΔCARD-mCherry in the presence or absence of zebrafish Asc-Myc, fixed at 48 h post-transfection, immunostained and imaged using a laser confocal microscope **(E,F)** or lysed, immunoprecipitated with ANTI-Myc Affinity Gel, and probed with antibodies to Myc and mCherry **(G)**. Representative views of maximum-intensity projection images of HEK293T cells are shown in **(E)**, quantitation of the percentage of Caiap specks in relation to the total number of Caiap transfected cells is shown in **(F)** and representative blots are shown in **(G)**. The sample size for each treatment is 300 for **(A,C)**, 30 for **(B,D)**, and 50 for **(F)**. Scale bar, 5 µm; ns, not significant; *p < 0.05; **p < 0.01; ***p < 0.001.

Although forced ubiquitous expression of *caiap* mRNA alone barely increased the resistance to ST infection (Figures 5D, 6A and 7A) and caspase-1 activity (Figures 5C, 6B and 7B), forced expression of *caiap* in macrophages, using the macrophage-specific promoter *mpeg1* (27), resulted in increased resistance to ST infection (Figure 5G). Notably, Caiap levels did not affect fish susceptibility (Figures 6A,C) or caspase-1 activity (Figures 6B,D) in response to a syngenic double ST mutant for SPI-1 and SPI-2, which contains a large number of genes encoding a type 3 secretion system that is required for virulence in mouse (45) and zebrafish (19), comparing with the controls. Moreover, the impact of Caiap on ST infection seemed to depend on flagellin, since the virulence of the flagellin mutant strain (FliC/FljB) of ST (35) was not affected by Caiap (Figures 6E,F), while the increased larval resistance and caspase-1 activity to ST which overexpresses flagellin (FliCON) (36) were found to be Caiap-dependent (Figures 6E,F). These results suggest that Caiap functions downstream of flagellin, although other PAMPs may also regulate Caiap activation.

### Zebrafish Caiap Requires Asc to Mediate the Inflammasome-Dependent Resistance to ST

Most inflammasomes require the adaptor protein ASC for their assembly, activation, and function. However, some NLRs that contain a CARD domain can bind directly to caspase-1. To this group belongs NLRC4, which has been shown to take part in host defenses against bacterial infection (46, 47). However, it has recently been found that ASC can strengthen the signal of the NLRC4 inflammasome (48) or being required for some specific responses (49). The presence of a CARD domain in Caiap suggests that it may directly recruit and activate Caspase-1 without the need for ASC, at least in most evolutionarily advanced species where true orthologs of mammalian caspase-1 with CARD domain exist. To check this, we ablated Asc using a previously validated translation-blocking MO (19). Genetic depletion of Asc in zebrafish drastically increased the susceptibility to ST of both WT and Caiap-deficient larvae (Figure 7A). In addition, Asc deficiency inhibited basal and Caiap-induced caspase-1 activity in non-infected and infected larvae (Figure 7B). Conversely, forced expression of Asc strongly increased larval resistance to the infection and caspase-1 activity, both effects being largely independent of Caiap (Figures 7C,D). Unexpectedly, however, reconstitution of Caiap and Asc complexes in HEK293T cells, which lack each of these components, showed that Caiap and CaiapΔCARD fused to mCherry were both diffusely distributed in the cytosol in the absence of Asc but formed a ring around the speck in the presence of Asc (Figures 7E,F). In addition, co-immunopreciptation assays confirmed that Caiap was unable to physically interact with Asc (Figure 7G).

### Zebrafish Caiap Is Required for Caspa Activation

**Figure 8:**
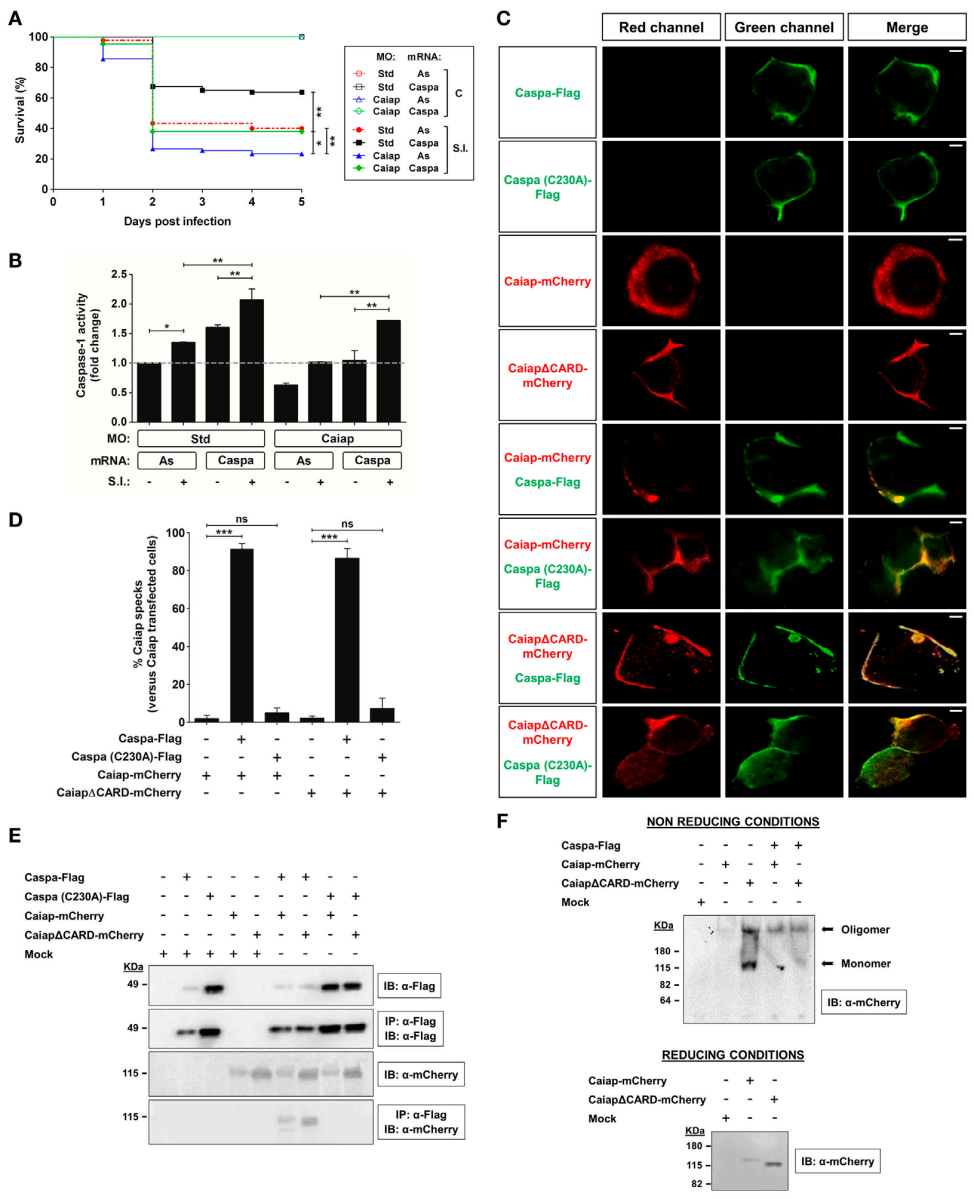
Caspa is required for the Caiap-mediated resistance to *S*. Typhimurium. **(A,B)** Zebrafish one-cell embryos were injected with standard control (Std) or Caiap MOs in combination with antisense (As) or Caspa mRNAs. At 2 dpf, embryos were infected with ST and survival **(A)** and caspase-1 activity **(B)** were determined as described in Figures 5A,B, respectively. S.I., ST infection. **(C–F)** HEK293T cells were transfected with zebrafish Caiap-mCherry or CaiapΔCARD-mCherry in the presence or absence of zebrafish wild-type FLAG-Caspa or catalytic inactive FLAG-Caspa (C230A), fixed at 48 h post-transfection and imaged using a laser confocal microscope **(C,D)**, or lysed and immunoprecipitated with ANTI-FLAG M2 Affinity Gel and probed with antibodies to FLAG and mCherry **(E)** or lysed and resolved under non-reducing and reducing conditions **(F)**. Representative views of maximum-intensity projection images of HEK293T cells are shown in **(C)**, quantitation of the percentage of Caiap specks in relation to the total number of Caiap transfected cells is shown in **(D)** and representative blots are shown in **(E,F)**. The sample size for each treatment is 300 for **(A)**, 30 for **(B)**, and 50 for **(C)**. Scale bar, 5 µm; not significant; *p < 0.05; **p < 0.01; ***p < 0.001.

Caspase a (Caspa, also known as Caspy) is considered the functional homolog of mammalian caspase-1, since it preferentially cleaves AcYVAD-AMC, a caspase-1 substrate, and interacts and co-localizes at the speck with zebrafish Asc in HEK293T cells (18). In addition, recent studies have demonstrated *in vivo* that Caspa is the effector enzyme of the zebrafish inflammasome in both macrophages (20) and neutrophils (19). Therefore, we next analyzed whether the forced expression of Caspa was able to rescue the high ST susceptibility of Caiap-deficient larvae. The results showed that Caspa was indeed able to rescue the infection susceptibility (Figure 8A) but only partially caspase-1 activity (Figure 8B) of Caiap-deficient larvae. In addition, forced expression of Caspa alone increased the resistance of larvae to ST infection (Figure 8A), confirming previous results (19).

The above results show that Caiap acts through Asc and Caspa to promote ST resistance, although this does not clarify the mechanism by which Caiap plays this function. We therefore reconstituted Caiap and Caspa complexes in HEK293T cells and, surprisingly, WT Caiap and CaiapΔCARD were both able to form specks in the presence of wild type but not of catalytic inactive mutant Caspa (Figures 8C,D). These results were further confirmed by pull down assays, in which WT Caiap and CaiapΔCARD were able to physically interact with WT Caspa but not with catalytic inactive Caspa (Figure 8E). In addition, while WT Caiap overexpressed in HEK293T cells self-oligomerized, assayed under non reducing conditions, CaiapΔCARD required active Caspa to fully oligomerize (Figure 8F). Taken together, these results show that Caiap interacts with catalytic active Caspa (P20/P10) through its ANK domain and self-oligomerizes *via* its CARD domain.

## Discussion

Although many recent studies have demonstrated the crucial role of the inflammasome as a molecular platform involved in the sensing of intracellular pathogens, little information exists concerning phylogenetic aspects of its composition, activation, and function. Analysis of genome databases has revealed (i) the enormous diversification of NLR in ray-finned fish (class Actinopterygii) (14); (ii) the existence of a single ortholog of the main inflammasome adaptor ASC in all non-mammalian species examined; (iii) that true orthologs of caspase-1, i.e., harboring N-terminal CARD and C-terminal caspase (P20/P10) domains, are restricted to the superorders Protacanthopterygii (trout and salmon) and Acanthopterygii (seabream, seabass, and medaka) of ray-finned fish (15–17), while most primitive Ostariophysi (catfishes and zebrafish) have a functional homolog of mammalian caspase-1, called Caspa in zebrafish, which harbors N-terminal PYD and C-terminal P20/P10 domains (18–20); (iv) the absence of a caspase-1 cleavage site in non-mammalian vertebrate IL-1β sequences (50); and (v) the absence of IL-18 in some ray-finned fish species, such as the zebrafish (16). Although the functional relevance of this extended array of NLR genes in zebrafish still needs to be investigated, recent functional studies have shown that the mechanisms of activation of the inflammasome are not fully conserved in ray-finned fish and that IL-1β is not processed *in vivo* by caspase-1 in this animal group (16, 19, 44, 51). However, these studies also demonstrated that the inflammasome is activated *in vivo* in zebrafish macrophages and neutrophils upon bacterial and viral infection, which leads to differential outcomes: pyroptosis of macrophage (20, 52) or PLA2-dependent biosynthesis of eicosanoids in the neutrophil (19). Together, these results support the idea that the use of the inflammasome as a molecular platform for the induction of pyroptotic cells death and the regulation of eicosanoid biosynthesis predates the split of fish and tetrapods more than 450 million years ago, while its use for processing pro-inflammatory IL-1β and IL-18 was later acquired in the tetrapod lineage.

To make this story more intriguing, we have identified a novel inflammasome component, Caiap, which is evolutionarily conserved from cartilaginous fish to marsupials, but unexpectedly absent in placental mammals. One of the most interesting observations of this study is the unprecedented combination of domains found in Caiap: an N-terminal CARD domain and several C-terminal ANK repeats. These domains are the regions of Caiap showing the highest degree of conservation at the primary structure level. In addition, 3D structure analysis predicts, with very high confidence, a strikingly similar tertiary structure of Caiap across all vertebrates, suggesting a conserved mechanism to regulate inflammasome activation.

**Figure 9:**
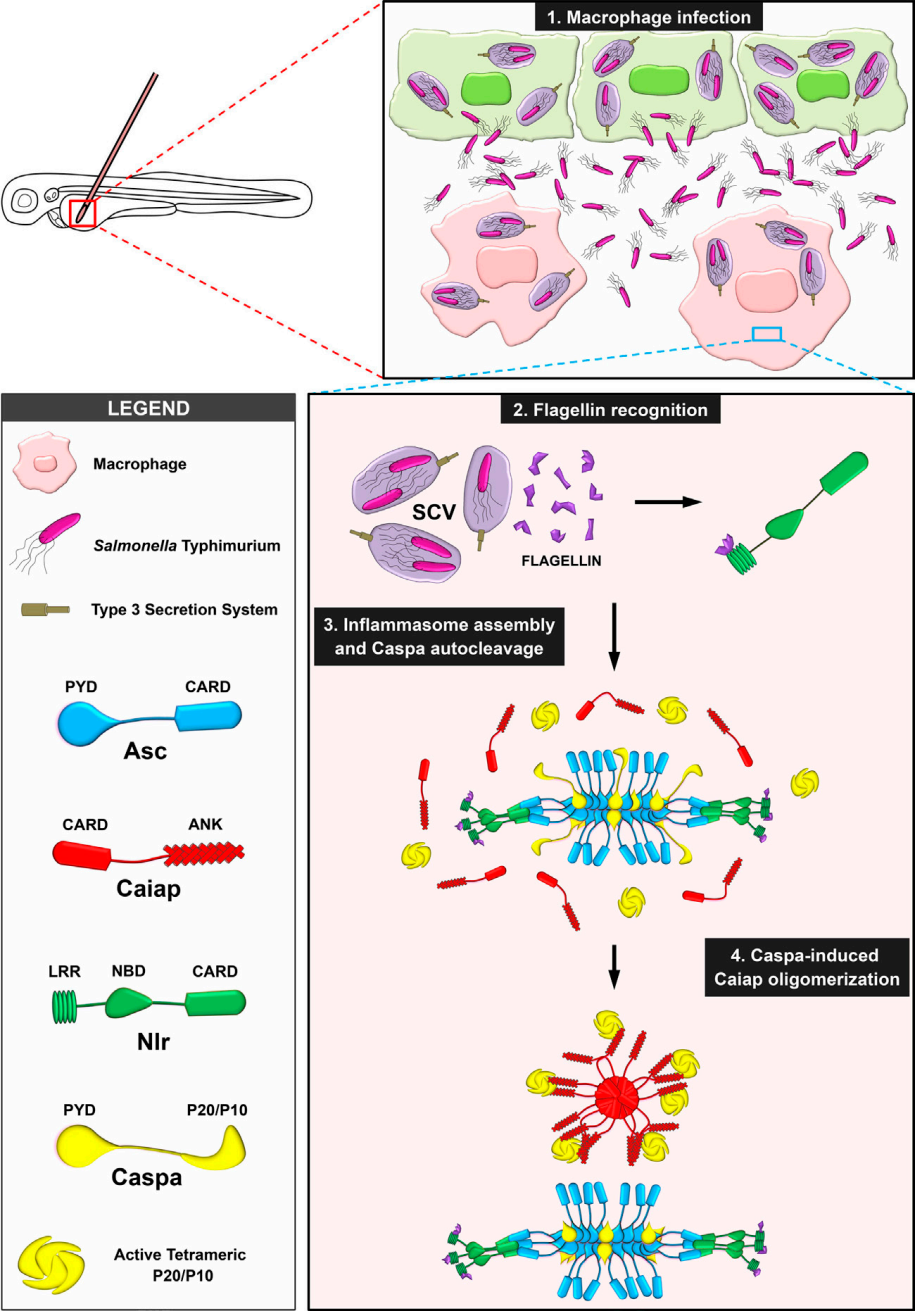
Proposed model illustrating the stabilization of catalytic active Caspa tetramers by Caiap. 1. Macrophages are recruited to the infection site where they are infected by *S*. Typhimurium (ST), which is contained within the *Salmonella*-contained vesicle (SCV). 2. Flagellin is inadvertently injected into the cytosol though the type 3 secretion system of ST where it is recognized by a Nlr. 3. The inflammasome is assembled *via* formation of Asc specks. 4. Active Caspa interacts with Caiap *via* its ANK domains and induces its self-oligomerization through CARD/CARD allowing Caspa stabilization in high molecular weight complexes upon its prodomain release.

Caiap is expressed at very low levels in zebrafish, but its expression is modestly induced by infection in immune cells recruited to the infection and wounding sites. Although the expression profile of Caiap requires further investigation, the induction of *caiap* in sorted macrophages from infected animals, together with the WISH data and the increased resistance of larvae forced to express Caiap in macrophages, suggests that *caiap* expression is restricted to a specific population of macrophages (53). Genetic experiments demonstrate that Caiap acts downstream flagellin and mediates its antibacterial activity through Asc and Caspa, as we have recently found for Gbp4 which, in contrast, is expressed in neutrophils (19). Another important difference between Caiap and Gbp4 is that forced ubiquitously expression of Gbp4 results in increased larval resistance to the infection (19), while Caiap overexpression barely increases infection resistance. This difference may be related to the different mechanism of action of each protein, since Gbp4 directly binds Asc through homotypic CARD/CARD interactions (19), while Caiap does not directly interact with Asc but with enzymatically active Caspa (P20/P10) through its ANK repeats (Figure 9). Strikingly, the CARD domain of Caiap allows its self-oligomerization, what seems to be essential *in vivo* to mediate the inflammasome-dependent resistance to ST. To the best of our knowledge, this is the first description of an inflammasome adaptor protein that directly interacts with the active effector caspase further promoting its activation probably by stabilizing caspase-1 in functionally stable, high molecular weight complexes after its autoproteolytic cleavage and prodomain removal (54). Caiap, therefore, is reminiscent of the inflammasome inhibitors INCA and ICEBERG that directly interact with caspase-1 but, in this case, *via* CARD domains (55–57), the former being able to cap and terminate the caspase-1 filament (57). Although the novel mechanism reported here also operates in other vertebrate species with orthologs of mammalian caspase-1 needs further investigation, the highly conserved primary and tertiary structure of Caiap across vertebrate species and the ability of zebrafish Caiap to interact with active Caspa through its ANK domains strongly support an evolutionarily conserved mechanism of action of Caiap.

The absence of Caiap in placental mammals suggests that Caiap was lost in this animal group after its split from marsupials. It is tempting to speculate, therefore, that other proteins replaced Caiap in placental mammals to carry out a similar function in the stabilization of the inflammasome. Strikingly, a recent interactome analysis of ASC complexes in human monocytic THP-1 cells has identified a protein called ArfGAP with GTPase domain, ankyrin repeat, and PH domain 3 (AGAP3) (58), which curiously harbors both GTPase and ANK domains. It is tempting to speculate, therefore, that Caiap and other ANK domain-containing proteins are involved in the stabilization of caspase-1 in functionally stable, high molecular weight complexes (54).

In summary, we report here the identification of a novel, evolutionarily conserved inflammasome component with a unique domain organization, which interacts with active effector caspase and is required *in vivo* for the inflammasome-dependent resistance to bacterial infection. This study supports the relevance of a broad evolutionary analysis of innate immunity mechanisms to understand the complexity of human immunity.

## Ethics Statement

The experiments performed comply with the Guidelines of the European Union Council (Directive 2010/63/EU) and the Spanish RD 53/2013. Experiments and procedures were performed as approved by the Bioethical Committees of the University of Murcia (approval numbers #537/2011, #75/2014, and #216/2014).

## Author Contributions

VM conceived the study; ST, SC, and VM designed research; ST, SC, AP-O, AV, DG-M, and FA-P performed research; ST, SC, AP-O, AV, DG-M, FA-P, MC, and VM analyzed data; and VM and ST wrote the manuscript with minor contribution from other authors.

## Conflict of Interest Statement

The authors declare that the research was conducted in the absence of any commercial or financial relationships that could be construed as a potential conflict of interest. The reviewer SB and handling editor declared their shared affiliation.

## Footnotes

^1^http://pfam.xfam.org/.

^2^http://www.uniprot.org/.

^3^http://www.bioinformatics.org/sms/index.html.

^4^http://smart.embl-heidelberg.de/.

^5^http://www.reading.ac.uk/bioinf/IntFOLD/.

^6^http://www.pymol.org.
