# Supplementary material for: Identification of an Evolutionarily Conserved Ankyrin Domain-Containing Protein, Caiap, Which Regulates Inflammasome-Dependent Resistance to Bacterial Infection
Source: Front Immunol. 2017 Oct 19;8:1375. doi: 10.3389/fimmu.2017.01375 (PMC5662874; doi:10.3389/fimmu.2017.01375)
Supplement: Supplementary file 1 [file table_1.pdf]

Table S1. Morpholinos used in this study. The gene symbols followed the Zebrafish Nomenclature Guidelines (http://zfin.org/zf_info/nomen.html). ENA, European Nucleotide Archive (http://www.ebi.ac.uk/ena/).

Gene	ENA or Ensembl ID	Target	Sequence (5’→3’)	Concentration (mM)	Reference
caiap	ENSDARG00000092758	atg/5’UTR	CTGGTTGAGCCCATGTCCAGTGCTT	0.16	This work
asc (pycard)	ENSDARG00000040076	atg/5’UTR	GCTGCTCCTTGAAAGATTCCGCCAT	0.6	Tyrkalska *et al.,* 2016
