# Supplementary material for: Identification of an Evolutionarily Conserved Ankyrin Domain-Containing Protein, Caiap, Which Regulates Inflammasome-Dependent Resistance to Bacterial Infection
Source: Front Immunol. 2017 Oct 19;8:1375. doi: 10.3389/fimmu.2017.01375 (PMC5662874; doi:10.3389/fimmu.2017.01375)
Supplement: Supplementary file 2 [file table_2.pdf]

Table S2. Primers used in this study. The gene symbols followed the Zebrafish Nomenclature Guidelines (http://zfin.org/zf_info/nomen.html). ENA, European Nucleotide Archive (http://www.ebi.ac.uk/ena/).

Gene	ENA ID	Name	Sequence (5'→3')	Use
rps11	NM_213377	F1	GGCGTCAACGTGTCAGAGTA	RT-qPCR
		R1	GCCTCTTCTCAAAACGGTTG	
caiap	NM_001025492	F2	AGCGCAGATATTGTTGCATAAGGGC	
		R2	GCCCCCACACGCAGTAGCAG	
gfp	EF591450	F5	ACGTAAACGGCCACAAGTTC	
		R5	AAGTCGTGCTGCTTCATGTG	
il1b	NM_212844	F5	GGCTGTGTGTTTGGGAATCT	
		R5	TGATAAACCAACCGGGACA	
caiap	NM_001025492	F	AGGCCATTGTGTATTTTCTGCT	Validation of gRNA
		R	AAGCTCATTGCAGCTATTGACA	
