# Supplementary material for: Identification of an Evolutionarily Conserved Ankyrin Domain-Containing Protein, Caiap, Which Regulates Inflammasome-Dependent Resistance to Bacterial Infection
Source: Front Immunol. 2017 Oct 19;8:1375. doi: 10.3389/fimmu.2017.01375 (PMC5662874; doi:10.3389/fimmu.2017.01375)
Supplement: Supplementary file 3 [file image_1.pdf]

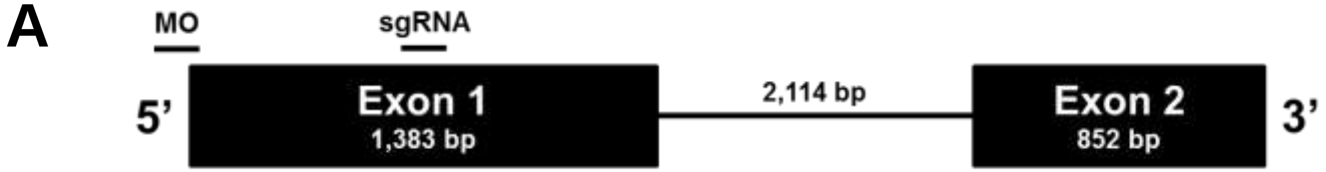

B

```
Tasmanian devil              ---------MCSSTHGQSSPYATEVMKMKKGELAAGIKDSDGLIQRLVDYGIFTAEQKLSLSYYKTNPEKVTRVLDMLISQGEHACRMFFYPCLKQLEPDLYHVIRKYTMGTEEVIPGAE  111
Gray short-tailed opossum    ---------MCSSTPGRNSPYATQVMKMKKEELAAGIKDADLLLKRLVDYGIFTSEQKLALSHYETHLEKVNRILDILISQGEHACQVFFYPCLKQLEPNLYQIIRKYTMGTDEIMYRTR  111
Large yellow croaker         MF-NLLKGFDAQATPNHINPYAVDVIRAKKMDLVHGISHTEDLLDHLVTEGVMTAAKKSIVLTIRTRQDQNSRVLDILEARGERACRKFFHPCLMLAEPKLYQEIKTYLSDVNGHIRDTR  119
Nile tilapia                 MW-NKVKGFESHAASNSKNPYAVDVIRTKRRDLVYGISHTEDLLNLLVAEGVMTTAKRSIVLSIRTHKDQNSRVLDILEARGERACRKFFHPCLMLAEPELYQRIKTYVGGMNEHVQDNR  119
Burton's mouthbrooder        MW-NNVKGFESHAASNSKNPYAVDVIRTKRRDLVYGISHTEDLLNLLVAEGVMTTARRSIVLSIRTHKDQNSRVLDILEARGERACRKFFHPCLMLAEPELYQRIKTYVRSMNEQVQDNR  119
Elephant shark               -------MVLMHPTGIFTNPYAIEVLQRKKSELVAGIANTDHLLDWLIENGLVSADKRLAVSSYRTREEKNARLLEMLVSRGERACRLFFYPCLKRVEPALYNQIRSYVSEVSGSFGDAR  113
Atlantic herring             ----------MSSTKTTVNPYAVEVIKTKKEELVGGILNTEELLDLLVDHGIISHDQRLLVSSIQTQEERNSKLLDVLISRGERACRIFFYPCLKRAEPDLYQNMRAYAGSVNEDIRDAR  110
Zebrafish                    -----------MGSTSFTNPYAIEVIQMKRSELVSGILNPEDLLDLLIANGVLQPDIRVLVSDLTVREEKNSRMLNILISRGERACRIFFYPCLKRAEPDLYQHMRTYVGGVNEGIRDAR  109
Blind cave fish              -----------MGSTKFTNPYALEVIRTKEKELVGGISNTEDLLDLLVANGVLLPESRVLVSCIAARDEKNSRMLSILVSRGERACRIFFYPCLKSAEPDLYQSMKTYVSGLNQNIKDTR  109
African coelacanth           ----------MSSRSLFINPYAIEVLKTKKQELIEGISNTDHLLDWLVDNAILTPDKKVLVSNYRTREEKNSRVLDILISRGERACRLFFYPCLKHVEPELYNNIRKYASSINVSVGDVR  110
Western clawed frog          MSTPATQLPAISSTSLFTNPYAVEVLKMKKQELVEGIKDPDTLLQSLIDNGIMLSDKKMVLSYYKTKTEKNSRLIDILLSTGERACRLFFHPCLKQVEPSLYNSVKNYVSTVNDSVGDGR  120
American chameleon           -----------MGSSIYTNPYAIEVLKMKKNELVEGINNPDHLLDCLIEYGIFTPEKKLVLSYYRTRRAKNSRVLDILASQGERACRLFFYPCLKEIEPYLYSQIRNYVSDVNVKIGDAR  109
King cobra                   -----------MYSSLFTNPYATEVLKMKKNELVEGIKNPDHLLDRLIEKGALTTEKKMILSYYRTRMAKNSRVLDILASQGERACRLFFYPCLKEVEPRLYSKIRNYVSDVNEKIGDAR  109
Common garter snake          -----------MYSNLFTNPYATEVLKMKKNELVEGIKNPDHLLDCLIEKSILSPEKKMVLSYYRTRMAKNSCILDILASQGERACRLFFYPCLKEVEPRLYSNIRNYVSNVNETVGDAR  109
Chinese alligator            ----------MHSTSLFTNPYAVEVLQTKKEELVEGINNPDHLLNWLITNGIFTPEKKMVMSYYRTRIEKNSRVLDILVSQGERACRLFFYPCLKQVEPNLYNSVRRYVSDINESIGDAR  110
Western painted turtle       ----------MYLNSLFTNPYAIEVLKTKKEELVEGLNNPDHLLNWLIDNGIFTPEKKMVMSYYRTRRAKNSRVLDILVSQGERACRLFFYPCLKQVEPNLYNRIKKYVSDVNESIGDAR  110
Green sea-turtle             ----------MYLNNLFTNPYAIEVLKTKKEELVEGIKNPDPILNWMIDNGIFTPEKKIVMSYYRTRIAKNSRVLDILVSQGERACRLFFYPCLKQVEPNLYNRVKKYVSDVNESIGDAR  110
White-throated sparrow       --------MIRHSSSLFTNPYAIEVLRTKKEELVQGINDPEQLLHWLVENGIFTPEKKLVLSFYRTRTAKNSRVLDILMSQGERACRLFFYPCLKQVEPKLYSKIRKYVSEVNESIGDAR  112
Chicken                      ----------MHSTSLFTNPYAIEVLRTKREELIEGINDPDHLLSCLIDNGIFAPEKRMVMSFYRTRTEKNARVLDTLISQGERACRLFFYPCLKQAEPKLYNKMRNYVSEVNESVRDSR  110
Turkey                       ----------MHSTSLFTNPYAVEVLRTKREELIEGINDPDHLLSCLIDNDIFSPEKRMVMSFYRTRTEKNSRVLDILISQGERACRLFFYPCLKQVEPKLYNKMRKYVSEVNESIRDAR  110
Budgerigar                   ----------MHSTSLFTNPYAIEVLKTKKEELIEGITHPDHLLNWLIDNGVFTPEKKMVMSFYRTRTEKNSRVLDLLMSHGERACRLFFYPCLKQVEPKLYSKIRKYVSETNESIGDAR  110
Golden eagle                 --------MIMHSASLFTNPYAIEVLRTKKEELVEGIKDPDRLLNWLIDNGIFTPEKKMVMSFYRTRTQKNSRVLDILISQGERACRLFFYPCLKQVEPKLYSKIRKYVSEVNESIEDAR  112
Barn owl                     --------MIVHSVSLFTNPYAIEVLRTKKEELVEGINDPDHLLNWLIDNGIFTPEKKMVMSFYRTRTEKNSRVLDLLISQGERACRLFFYPCLKQVEPKLYSKMRKYVSEVNESIRDAR  112
Spotted gar                  ----------MHSNRLFTNPYAIEVLQTKKRDLIEGISKTEHLLNLLIDKEVLPPEKRLLVLSCRSREEKNSRVLDFLVSQGERACRLFFYPCLKLIEPDLYNSIKVYVGDVNESIGDGR  110
Asian bonytongue             -----------MHSAIFPNPYATEVLHLKKKDLIEGISKTEYLLNLLIDKGVFPPEKKALMSYDRTREEKNSEFLDVLLSQGERACRLFFYPCLKLVEPDLYQSIRTYVGEINKNVRDAR  109
                                                  .*** :*:: *. :*  *: . : ::  ::    .    :  :     .  :    .:. * : **:**: **:***   ** **  :: *     .  .   .
```

```
Tasmanian devil               GQLVGHLLDRDKESLEKSTKLDHEKQNTGIQEAS----VK-PKTISFTA-K---QRKFPPIQTPVNSPTPPIYLLTASGKGDVADTENVLEDKHMGEVTFPRESPLGMAMADEPTELLGF  222
Gray short-tailed opossum     QQSVGHFLDGNKNGLEKSAEKIQKKHNTMIQAAS----VK-PKLVSYLAAK---TKGYPASQTPRNSPTSPIHALNASGKGDVD-VENGLENRHGCKVMSPRENPLGAAVAKSLDEIHNY  222
Large yellow croaker          RQLIGYLLERDQEGIDKITSKVVSQET-----PILFAI-KEPKKSLPEKEDRSTVPLLTPEEHEPAQSRAENFIHMIATDGEVILLEELLENTDINTVNSSNETLLHVAAQYGHLSIIEL  233
Nile tilapia                  RQLIGYLLERDCDWGGNLTDEIGTQRT-----YT---N--I-KKRSSVTKKGSGTIVLATSEHTPAQSKSESLIYMAARKGDLLLLEELLKDSDINTVNSSKETLLHVAAENGHLPITEL  228
Burton’s mouthbrooder         RQLIGYLLERDCEWGGKLTDQTGTQRT-----YT---K-RI-KKGSSVTKKGSGTIVLATSEHTPAQSKPESLIYMAARIGDLLLLEELLKDSDINTVNSSKETLLHVAAENGHLSIAEL  229
Elephant shark                RQLVGYLLERDKDEVPRSRRQREEEEE---VAPSLR----------PVKPKPRE---------P-VKPRDTEELYRAAAQGDLPGLERALQDADVNAINASNETLLHVAAAHGHAVMVSY  210
Atlantic herring              RQLIGYLLERDHQRPFAAANTQNST-------FKKTSLKTPSKVSKKHEAKNAEM----KEWEPLEPEQQPSHIFTAIANGDLSLLEEFLVGKDVNSIRQGSDSLLHVAAEDGQLSIIEL  219
Zebrafish                     RQLIGYLLEKDKQGLVKNSKPERIH-------PKS-TANE---P--NNEPL--NK----QILK--SESNHHDAILEAISSGDLYLLQELTKELDVNSVLSSNDTLLHHAAEYGKEAIVYF  208
Blind cave fish               RQLIGYLLEKDCKGSKIKKKINTDH-------SNE-TLNHESKR--AEEPV--SS----EGLEKNSTKLTSKDILKAVSTGDLSLLQALLKETVISDRNSSIDTLLHIAADHGQVAVINF  213
African coelacanth            RQLVGYLLEKDKDGIPKSRQENVIKTTS--------KIIPSR-KGSTIIRKDTEKSI-PPASKPKGDSSKTDNIYEAVATGNLSYLEEVLKINNINEANTLNETLLHIAAANGHISVIQY  220
Western clawed frog           RQLVGYLLERDKDVPKTIIKDRPHKAQQRESLPKKVKALDEK-PSYSEKEKKSVPQQ--ISKDVKRTDSSSMTVFDAVTKGDLSILRNILRDTDINAVNPSGETLLHIAAASGHVAVIEY  237
American chameleon            RQLIGYLLEKDKGWTQTDKDL----QQEKKDSPRQSLVKPQK----LVR-------------VKHKSKQSPNVFDSVAKGNLSELEKYLKENEVNAVNSANETLLHIAAAHGHTEIIDY  207
King cobra                    RQLVGYLLEKDKGCIQKDKAS----HQGGKDSPRSI--KPKK----MIYDKPKSKST-LA---ARQSLDNVPTVFDLVRKGILSDLEKSLNPTDVNATNSVNETLLHIAATHGHLEIIDY  215
Common garter snake           RQLIGYLLEKDKGSIQKDKAS----HQGKQASPRSI--KPKK----MIYNKPKSKST-LA---ARQSLDNVPTVFDLVRKGTLSDLEKALNPSNVNATNSANETLLHIAAAHGHTEIIDY  215
Chinese alligator             RQLVGYLLEKDKDWIEKSKEQ----HKEKTDIPRQIKRK--R----GIIEKVKETQS-LPAAKTKKDHSDTVSIFGAAAKGDLSNLEKMLKENDINAVNSSNETLLHIAAANGQIAVMEY  219
Western painted turtle        RQLVGYLLEKDKEWIETTSEQ----NQEKKDIPRQILSKQER----ATKEKVKQTQN-LPAAKPKKDHSNAVSIFDAAAKGDLSDLAKVLKENDINAVNASNETLLHIAAANGHLAIIEY  221
Green sea-turtle              RQLVGYLLEKDKEWIETTSEQ----HQEKKDIPRQILSKQER----ATKEKVKQTQN-VPAAKPKKDDSNAVSIFDAAAKGDLSDLAKVLKENDINAVNASNKTLLHIAAANGHVAIIEY  221
White-throated sparrow        RQLVGYLLEKDKVWFENSSEQ----HQGNNDRPGGKK--HKG----ALKKKGKVTLL-SRATKPRNDPA-AVDIFAAVAKGSLPELEKTLKDNDLSVLNPSSETLLHLAAAHGHLSIVAY  220
Chicken                       RQLIGYLLERDKVWFEKSYEQ----CQEERESPGRKR--HEG----DTKKKGKETQL-SGAAKPREHHS-DIGIFDTAAKGYLSELEKTLKDNDINALNSSNETLLHVAASQGHLSIMEY  218
Turkey                        RQLIGYLLERDKVWFEKSCEQ----YQEERESPRRKR--HEG----ATKKKGKETQL-SGAAKPRKHHS-DIGIFDTAAKGYISELAKTLKDNDINALNSSNETLLHVAASQGHLSIMEY  218
Budgerigar                    RQLVGYLLEKDKVWFESSNEW----HQGKKDCLRRKT--KEW----AIRKKGKKLQL-SRTAKPRKDHT-DVDIFDAVTKGNLSELEKTLKDNDINKLNPSSETLLHVAAANGHATVMEY  218
Golden eagle                  RQLVGYLLEKDKVWFENSSEQ----HQEKKHSPRRKN--QEQ----AIKKKGKETQL-SRAAKPRKDHA-DVGIFDAVAKGYLSELERTLKYNDINALNSSSETLLHVAAANGHLTIMEY  220
Barn owl                      RQLVGYLLEKDKVWFENSSEQ----HQGKKDTARRKK--QKR----TIKKKGRETQL-SRAAKARKGHA-DIGIFDAVAKGYLSELEKTLRDNDINALNSSSETLLHIAAANGHLTIMEY  220
Spotted gar                   RQLIGYLLERDHSNPPKNTQHKIPEKTTNMAVPRKRVKIQDSKPVSTIS------SL-KTPEETEVVHRKPGGLFDSAAKGDVSCLEKLLSDCDINAVNSLNESLLHIAALNGQVPIIEF  223
Asian bonytongue              RQLIGYLLERDKEEPRVHNSDFTPPNNVYSAAPLKKVISQANKKENNFQPTT--ISK-ANVGAAKKMQHNHNGIFDLVATGKLSLLEELWKGKDVNAINSSQETLLHIAAQHDQVAVMKF  226
                               * :*::*: :                                                                     * :                   .. *  *       :

Tasmanian devil               PIAKGPRAEGKEGKEKSLLQRAAERARGDAVKI-LQGGMGSSNLERDIMMPLGFNLQ-GSQLSLIKPILEENIG-----TPYYFQHRSVFQEEYEGFRVFPDRDKLLDAMEVHKDHRADP  335
Gray short-tailed opossum     SMIKSFKAEGKDGKEKSLLQRAAERARGDAEKV-LQGGMSGNNLDS--RVPMGFHVQ-GGKHTVMRLMLERNPG-----NPYYLQYRPALQAEDQGVQVCLEDDNLLNAMD-----KADT  328
Large yellow croaker          LIRKGARLDLQDNKGHTALHRAASRGHTEIVRALVQAGAPIYTLDLQGKTSVHLAAE-NEHLHPVKVLVKEEAKQSESHTRDMFLHSAAVEDNWRLAEILLQSGADVDARNNH----KKT  348
Nile tilapia                  LIRKGARLDLQNDAGHTALHRAASRGHTELMKALVKAGAPIHNLDLKGKTPIHLAAE-NRHLKSVKLLVEEEARQSESNRQNMFLHMAAMEDDWRLAELLLQSGATVDALNSH----NKA  343
Burton’s mouthbrooder         LIQKGARLDLQNDAGHTALHRAASRGHTELMKTLVKAGAPIHTLDLKGKTPIHLAAE-NRHLKSVKLLVEEEARQSESHRQNMFLHMAAMEDDWRLAELLLQSGATVDALNSH----NKA  344
Elephant shark                LLSQGAKMEARDSKGRSPLHRAAEEGQAEAIRALLAAGAHIYAMDSDSQTPLHLAAPHQSHSAVVKLMLREEGKRAD--RKSSFLHMAAQRDESGFARTLLRHGAPVDAKDGR---RHQT  325
Atlantic herring              LLQSGAKLDARDHQGRTALHRAAGKGHTAAAMSLVMAGADIYICDNVSKSPLHLAAQ-NGHQDTVQALVKEECRYLK--KHTTFLHMAAVQDDSELAEVLLRAGAEVDCKDSC----RKT  332
Zebrafish                     LLRQGAKLDLKDKEGRTALHRAAQRGHTAVAVALAKAGADIHATDQTSKTPLHLAAQ-NGHEGCVKALVHEEKKSLK--NQTTVLHMAAIEDNATLAEVLLRNGALVDAQDGQ----RKT  321
Blind cave fish               LLRKGAKLDMRDHQGRTALHRAAEAGHMAASMALVRAGADIHAKDQASKTPQHLAAQ-NGHRNTVKSLIAEEAKSCK--NQTTFLHMAAMEDDPVLADILLKNGASADAQDSQ----RKT  326
African coelacanth            LIEKGTKTDVRDSKGRSPLHRAAENGHVNAVKTLLQAGADIYAFNKESKAPLHVAAQ-NSHFSIVKILLDEEARVYK--NRKNFLHLAALKDDSNLAQILLSNGAPVDAKDDE----KKT  333
Western clawed frog           LINKGAKIDCKDIKHRTPLHRAAENGHGEAVKVLLRAGAFIYSLDDDSLTPLHLAAE-NNHQNVVKILLQEEGRQYK--NRHNFIHMAATQGNNKLMQLLLKNKAPVDAVDEK----SQT  350
American chameleon            LISKGAKLEVKDQKERTPLHRAAKEGHGKAVNMLLQAGADMYTLAQEGKTPLHLANQ-NNHTHILKSILEEEARRHK--NQHNFLHMAALRNDSDLVKMLLKSGALLEAKDER----GQT  320
King cobra                    LISKGAKLEVKDNKGRTPLHRAAEKGHDKAVNRLLQAGANMYSLDQEGKTPLHLANQ-NQHTHILRNILKEEARRHK--NQHNFLHRAALKDDSDLVQVLLKNGALVDARDER----GQT  328
Common garter snake           LISKGAKLEVKDNKGRTPLHRAAEKGHAKAVNRLLQAGANMYSLDQEGKTPLHLANR-NQHTDVLKNILKEEARRHR--NQHNFLHMAALKDDSDLVQVLLKNGALVDARDER----GQT  328
Chinese alligator             LIKKGAKLEVKDSQGKTPLHRAAEKGQDDAVKVLLQAGAYIYSLDEAAKTPLHLAAQ-NHHGPTLNRILKEEAKRYR--NQHNFLHMAALKDESSLAQMLLKNGASVDAKDEK----GQS  332
Western painted turtle        LINKGAKLNVKDKKGRTPLHRAAEKGQDDAVKVLLQAGAYIYSLDKEAKTPLHLAAQ-NHHTHILKRILKEEARCYK--NQQNFLHMAALKDESNLAQMLLKNGAPVDAKEEK----GQT  334
Green sea-turtle              LINKGAKIDVKDKKGRTPLHRAAEKCQDDAVKVLLQADAYIYSLDKEAKTPLHLAAQ-NHHTHILKRILKEEARCYK--NRQNFLHMAALKDESNLAQMLLKNGAPADAKDEK----GQT  334
White-throated sparrow        LLSKGARTGVKDSKGRTALHRAAQKGHAGAVRLLLRAGASMHTLDVEGKTPLHWAAE-NHHSHIVRMLLKEEARSCR--NQHTFLHMAALRDESSLAKMLLEAGASTERKDER----GQT  333
Chicken                       LISKGAKLDVMDQKGRIPLHRAAEKGHGDAVKVLLRRGAHMYSLDKEGKTPLHLAAQ-NNHSHILKMFLREEARRSG--NQQHFLHMAALKDESSLAKMLLKCGASIDGKDER----GQT  331
Turkey                        LISNGAELDVMDQKGRTPLHRAAEKGHGDAAKVLLRCGAHMYSLDKEGKTPLHLAAQ-NDHSHILKMFLREEARSCG--NPHHFLHMAALKDESSLAKMLLKSGASSDGKDER----GQT  331
Budgerigar                    LISKGAKVDVKDKNGRTPLHRAAEKGHGDAVKALLQSGAYLYSLDMKGKTPLHLAAQ-NNHSHIVKILVEEEARSYK--NQHNFLHMAALKDESSLAKMLLKAGASIDGKDEK----GQP  331
Golden eagle                  LISKGAKIDVKDKKGRTPLHGAAEKGHGDAVKVLLQCGAYMYGLDMEGRTPLHLAAQ-NNHSHILKMLLKEEARSYR--NQRNFLHMAALKDESSLAKMLLKAGASADEKDER----GQT  333
Barn owl                      LISKGAKTDVKDKKGRRPLHRAAENDRGDAVKMLLRCGAYMYSLDTEGKTPLHLAAQ-NNHSHIVKMLLKEEARSYR--NQHNFLHMAALKDESSLAKMLLKAGASTDGRDER----GQT  333
Spotted gar                   LLRKGVKMDLRDRKGRTALHRAAENGHSMAVKSLVQAGANIYAMDKESKTPLHLAAQ-NKHLNSVKILVEEEIKICH--NQRTFLHMAALKDNSHLAQILLQEGAPVDAKDNQ----KKT  336
Asian bonytongue              LISKGAKLDIRDSQGRTALHRAAEMGNTSATKVLLQNGADIYALDKYSDMPLDLAVQ-NIHQSTVKHIVEEEIRHHK--NRRTFLHVAAIKNNYNLVTVLLKSGSPVDAKDNQ----RKT  339
                               :.. .    :   :  *: **   .                                 :   :. :: .:           . :  . . :              :  :       .
```

```
Tasmanian devil             ASKSPEINSLDEAIKILLTAGINAD--SSIVHNAFHHDDQDVIQLLLKYSAA-LPRETRELALFEGIRKNLPEVVAALADGGTPLDVQNEHYYTPLLLAAEMDRTLCAEVLIQRGSNMEA   452
Gray short-tailed opossum   ASTFPGTNSLEEAIKILLTTGINAD--SGILHNAFHHDDQVVIQLLLKHSSI-LPSETKEQALFEGIRKNLPRVVTALVDVGTNLNARDHNQYTPLLLAAEMDRAACANVLIRRGANMAA   445
Large yellow croaker        ALFYAVSRNNVKTVTVLLNAGAKVD--YDVINEAIKLNEESILHLLLDNARGS----LREETLFSAVQQNHDGVVAALIDSGADVNMSDKQGYTPLLLSAALGHAEVFRVLVAKQAKLDA   462
Nile tilapia                ALFYAVKESNEKTMTVLLNAGAKAD--YDVINEAINLHQESILQLLLANARGVLSKDVLGSALFAAVRKNFDGAVSALIDSGADVNMRDGRRYTPLLLSAELGHSEVFSVLVAKNAKMDA   461
Burton’s mouthbrooder       ALFYAVKESNEKTMTVLLNAGAKAD--YDVIYEAINLHQESILQLLLANARGVLNKDALGSALFSAVRMNFDGAVSALIDSGADVNMRDGRRYTPLLLSAELGHSEVFSVLVAKNAKLDA   462
Elephant shark              PLFYAVSRGHINTAKVLLEAGAQVVDGPGLIDAAFSTGDQALIALMLTQARE-----VGPATIFRAVERDLPKVVAALAEMGADVNSRDGSGRTPLLMAAERGMAECATVLLEKGAQLRD   440
Atlantic herring            PLCYAVSRGNERTVAVLLQAGAQVD--SDAMASAFDLNSKSMLRLLLQNAPGQMTAESIHSALFRAVERNLGGIITALIDSGADVNTQNNQGYTPLLLAAELGNVEAFKALVGKKANLEG   450
Zebrafish                   ALHHAVRHGNEKTAAVLLKAGAQVD--SRIVDAAFQLNRKSLLSLFLQHVHESMSQTEINAALFKAVQRNLDAVVAALIEHGADVNSCNELGYTPVLLAAELGNGEAFKVLVSKKAKLDE   439
Blind cave fish             PLFYAISCGHEKTAAVLLQAGAQVN--SAIIEAAFDLNRESVLSLLLRNIKNRMSQNELKTILFKAVKRNLDGVVAALIEIGIDVNVRDDLGYTPLLLATELRNVEVFKMLVSEKAHLDK   444
African coelacanth          ALFHAVSHAFENTVKVLLEADAYVN--SSIIDAAFNSNNQSIFSLIMQYAKG-MSHATMVSALFKAVQKNLYGIIASLIDKGIKVNVRNEMEFTPLLLAAEMGKTEAAKILIEKGAKLDE   450
Western clawed frog         ALLYAVSGGHLKTVKMLLEAGASID--SSIIDAAFATNNERIFGLLLEYSKG-LSPDTMVSAVFKAVPLNLYGIINALIDKGTNINATNDIQYTPLLLAAELGKTESAQALIEKGAQLDV   467
American chameleon          ALGYAVSRGFKKTVKVLLEAGAKVD--SSIIDVAFNSNNQSLFRLLLEYANK-MSPETMVSALFKAVEQNLHGIVAALIERGIDINAHNEMQYTPLLVACEMGKMESAKILIEKGASLKE   437
King cobra                  ALSYAASQGHEKTVKVLLEGGAKVD--SSIIDAAFNSNHQSLFKLLLEYARG-LSSEAMVSALFKAVEKDLHGIVAALIERGTDINVRNEEQHTPLLVACKTGKIKSAKVLIEKGANLKD   445
Common garter snake         ALSYAVSQGHEKTVKVLLEGGAKVD--SSIIEAAFNSNNQSLFKLLLEYARE-LSSESMVSSLFKAIEKDLHGIVAALIDRGTDVNVRNEEQYTPLLMACKTGKIKSAKVLIEKGANLKD   445
Chinese alligator           ALSYAVSRGFETTVKVLLEAGASID--SNVLDVAFDSNNQSIFQILLEYFKG-LSPDTMVSALFKAVQKNLHGIVAALVDKGTDINVYNEMGYTPLLMACELGKTESAEVLIEKGASLKE   449
Western painted turtle      ALGYAISQGFEKTVKVLLEAGASID--SNIIDVAFNNNRQSIFRILLEYSKG-LSPDMMVSALFKAVQKNLHGIVAALVDRGTDINAHNEMQYTPLLMACEMGKTETAEVLIAKGASLEE   451
Green sea-turtle            ALGYAISQGFEKTVKVLLEAGASID--SNIIDVAFNNHKQSIVRILLEYSKG-LSPEMMVSALFKAVQKNLHGIVAALVDRGTDINAHNEMQSTPLLMACEMGKTETAEVLIAKGASLEE   451
White-throated sparrow      ALSYAVSQGSENTAKVLLEAGATVD--SNTVERAFNSNHPSIFKILLEYSKD-LPADIMELALFRAVQKNLHSVVAALIDRGTDVNAHNKKHYTPLLLACEMGKAESAEVLMEKGANLGI   450
Chicken                     ALSYTVHQGFENTAEVLLEAGASVD--SNVVERAFNSNHPSIFKTLLEYSKD-LPSDIMESALFKAVQKNLHGIVAALADRGTDINAYNEMQYTPLLLACETGKAECAEVLIEKGANLGI   448
Turkey                      ALSYAVSQGFENTAKVLLEAGASVD--SNVVEKAFNSNHPSIFKTLLEYSKD-LPLDIMESALFKAVQKNLHGIVAALADRGTDVNAYNETQYTPLLLACEMGKAECAEVLIEKGANLGI   448
Budgerigar                  PLSHAVSHGSENTAKVLLEAGASVD--SNITETAFNSNHPSIFKILLEYSKD-FPSEVMESALFRAVQKNLPGIVAALIDRGTDIDALNHMRYTPLLLACETGKAEAAEVLITKGANFGM   448
Golden eagle                ALSYAVSQGSENTAKVLLEAGASVD--SNMAERAFNSNHPSIFKLLLEYSKD-LSSDIMESALFRAVQKNLHGIVAALIDRGTDINAYNEMQYTPLLLACETGKAESAEVLIEKGADFSI   450
Barn owl                    ALSYALSQGSENTARVLLEAGASVD--SNMAERAFNSKHPSIFKTLLEYSKD-LSPDIMESALFRAVQKNLHGIVAALIDRGTDINAYNEMQYTPLLLACDTGKAESAEVLIEKGANFGI   450
Spotted gar                 ALFHAVHSGFEKTARVLLEAGAKLD--SSIIEAAFNLNSQSMYSLLLQHAKG-LSPDTMVSALFKAVQKNLQEVIAALIDRGTDINARNGMQYTPLLLAAELGNLEAVKILVAKKASLDE   453
Asian bonytongue            SLFHAVNLRNENTARVLLEAGAKVD--SDIMEAALNLNDKSMFSLILNYAKE-LPPDAMKSLLFKAVQRNLEWIIAALIDAGTDVNSRNDLQYTPLLLAAELGNVYAFNILMSKNASVED   456
                                        :  :**               *:      :   ::      .        :* .:  :    : :* : *  ::  :    **:*::           *: . : .

Tasmanian devil             KTSYAESALHLAVKAGGIYTVELLLESGMSPNVQGFNGQTPLHVAAWYNKHEMVGLLVQAGAQINILSTE-QNTPLHIASEKGNVDAAIQLIQYKADPNLKNKLNMTPLHLAAR-AGNKT   570
Gray short-tailed opossum   RTAHLDSALHLAVKAGAVYTVELLLDNGMSPNLAGWNGQTSLHTAAWYNKHEMVNILINAGANINALTTE-QNTPLHITSERGHVDTAIQLIQQAADLNMKNKLSMTPLHLAAR-AGNMT   563
Large yellow croaker        TLSDLSSALHLAVQSGSVSIVQTLLERGLDPNITGPKAQTPLHFAAQCNRPEMVDVLLRAGAKVNAVAQD-GLTPLHIASQQGHADSVTQLLQGKADPSIKDRLGRTALHWAASSKGEGR   581
Nile tilapia                TLSDLSSALHLAARSGSKPIVQTLLEKGLDPNIKGAKGHTPLHLAAQCDRPDITGLLLKGGAQVNAVSQD-GLIPLHIASRQGHTDTVIQLLHNKAEPGVKDRLGRTALHWAASSQVDSC   580
Burton’s mouthbrooder       TLSDLSSALHLAARSGSKPIVHTLLEKGLDPNIKGAKGHTPLHLAAQCDGPDITGLLLKGGAQVNVVGQD-GLTPLHIASMQGHTDTVIQLLHNKAEPGVTDRLGRTALHWAAFSQVDSC   581
Elephant shark              RAPDLSSALHLAVMSDSPSLVHLLLEKGMDPNITGAGEQTPLHLAARHDRYKMVDVLIGAGSKPNSAAVL-GATPLHVASRWGSRQAAERLIANKADINAKDRDARRPLHLAAL-VGDTP   558
Atlantic herring            RLSNSTTALHLAVQSGNVTITQVLLEMGIEPNITGPRDQTPLHMAAMHNQSALVGLLLRAGAQVNAITQD-GHTALHLASQRGHSEVVTQLVRAKADLSVRDKEGRGALHWAAA-QGEAS   568
Zebrafish                   RLPNQMSSLHLAVQSGSIQIAQILLHKGIDPNISGPKDQTPLHLSASHNQPAMMALLLRVGAQLNPVTQD-GFTPLHLASQNGHTEAVAQLLEAKADVHAKDKQGRTALHWAAE-QGEVA   557
Blind cave fish             KLPNQTSALHLAIQSGSMQITQILLNKGMDPNITGPKDQTPLHLSALYNQPTLMALLLRKGAQVDAVTQD-GLTALHLASQCGHTEAVAQLLEGKADVHAWDRQSRTALHCAAA-QGEAG   562
African coelacanth          RLPNQNTALHLVAQGGNLSVAKLLLENGLNANIVGSGDQTPLHVVAFHNKPAVADILIRAGAKVNAVTKESSVTPLHIASQRGSFSAAQCLIQHRANVKAKDKHSKTPLHFAAA-VGDYP   569
Western clawed frog         RTPNMSTALHLAVQGGDASITKLLIRKGININIAGPGDQTPLHVAAFHNKQELADILIAAGANVNVVTKE-LFTPLHIASQRGNLHVAQSLLHHKANVNAKDKQSRTPLHLAAE-GGAYE   585
American chameleon          KTPDSSSALHLAVQAGSTSIAKMLLHKGMDPNIMSQRDQSPLHVAAFHNKGALVDVLIEGGAKIDPVTKE-LHTPLHLASYKGHTEVAQKLLQNKAKVNLKDKQSKTPLHLGAE-KGHIA   555
King cobra                  KAPNLSSPLHLAVEAGAFSIAQMLLQKGIDPNITAQGNQTPLHVAAIYNRGALVDLLIEGGAKIDAVTTD-LFTPLHMASDKGHTDVALKLLQHKANVHLKNKRSKTPLHLAAD-MGNPA   563
Common garter snake         KAPNLSSPLHLVVEAGAFSIAQMLLQKGIDPNITAQGNQTPLHVAAIYNRGALVDLLIEGGAKIDAVTTD-LFTPLHLASEKGHTDVALKLLQHKANVHLKNKRSKTPLHLAAE-MGNPA   563
Chinese alligator           KTPSSDTALHLAVQAGAVAITSLLLRKGMNANITNQGDQTPLHIAAFHNKEALVDILINAGAKINAVTKE-LVTPLHIASQRGNADVAQKLLQHKANVNVKDKQSKTPLHLATE-LGDCA   567
Western painted turtle      KMPNSNTVLHLAVQAGAVSITNLLLRKGMNANITSQGEQTPLHVAAFHNKGSIVDILINAGAKINAVTKE-LVTPLHVASQRGNVDVAQQLLNHKANVNVKDKQSETPLHLAAE-KGDHA   569
Green sea-turtle            KMPNSNTVLHVAVQAGAVSITNLLLRKGMDANITSQGEQTPLHVAAFHNKGSIVDILINAGAKINAVTKE-LVTPLHVASQRGNVDFAQQLLHHKANVNVKDKQSKTPLHLAAE-KGDHA   569
White-throated sparrow      RTPAADTALHLAVQAGAAAIAALLLSKGMDANLRNQAEQTPLHVAALRNHGALVGLLLSAGATINAVTKD-SATPLHIASHRGHADAARQLLHHKAPVNAQDKQAKSPLHLAAE-QGHRT   568
Chicken                     KTPASDTALHLAVQAGAASITNLLLHKGMEANVTNQADETPLHIAALHNSGALVGLLVSAGAKINAVTKE-FVTPLHTASQRGNADVALQLLHHKANVNAKDKQSKSPLHFASE-RGDKT   566
Turkey                      KTPASDTALHLAVRAGAASITNLLLHKGMEANVTNQTDETPLHTAALHNNGALVGPLVSAGAKINAVTKE-FVTPLHTASQRGNADVAKQLLHHKANVNAKDKQSKSPLHFACE-RGDKT   566
Budgerigar                  RTPASDTALHLAVQAGAASTAHLLLHKGLETNLRNQAHETPLHIAALHNKGALVGLLVNAGAQINAATKE-FVTPLHIASQKGSTDAAQQLLYHKAHVNVKDKQLKSPLHFAAE-RGDRT   566
Golden eagle                KTPASDTALHLAVQAGAASIANLLLHKGIEINLMNQANETPLHIAALHNKGALAGLLVNAGAKINAVTKE-FVTPLHIASQRGNTDVAQQLLYHKANVNVKDKESKTPLHFAAE-RGDKA   568
Barn owl                    KTPASDTALHLAVKAGAASIANLLLHKGMEVNLMNQAHETPLHVAALHNNGALVGLLVNAGAKTNAVTKE-FVTPLHIASQRGNTDVAQQLLHHKANVNVKDRQSKSPLHFAAE-RGDKA   568
Spotted gar                 KLPNHNSALHLAAQSGNLPVLKLLLEKGMDVNMPGPVDQTPLHLAALHNKPDVVEVLIRHGAQVNAVTKE-VLTPLHIASQQGHQEVVEKLIQFKADINAKDKQSRTPLHLAPA-QGGAS   571
Asian bonytongue            KLPNGNSSLHLAVQSGNMHITKLLLEEGVDANAVGSQEQTPLHLTALYNQSALVEELLHVGAHINAVDQK-GLTPLHVASQQGHPDIVSLLIRREADISAKDKQARTALHWAAA-QPQAT   574
                                  : **:.  .        *:  *:. *      .: **  *  :   :   *:  *:  :          ** :*  *    .  *:   *     ::     **
```

```
Tasmanian devil             MVELLIHSGSDPNTADKEKKTPLHWASSEGHLEVVKT-MLIHKVRFGAKDMDGFSPLHYAALKGNVEMVKLFLEAGKNKNINERNIYRKTPLHLAAEQGHGDLIKLLLSCGAAVNALDNN  689
Gray short-tailed opossum   MVELLLHSGADPNVPDREKKTALHWAASASFIKVVKY-LLNYKARSGTRDMDGYSPLHYAALKGNVELVKIFLEAGKNKNINERNIYRKTPLHLAVEQGHTELIKMLINNGAAVNALDNN  682
Large yellow croaker        VVDLLLSAKANPNATDNEKKVALHLAAMEGKVDVVTS-LMSHKAKGGAKDMDGSTPLHYAAAGGHANVVSALLRLLNNKGVDERNTWRKTPLHAAAEKGHDSVVVLLLEAGAKINNTDQG  700
Nile tilapia                VVDLLLSAKADPDATDNEKKTPLHLAAMEGKVDMAIS-LLSHRAKRRARDMDGSTPLHYAAAGGHVSVVTALLQPLNNKGTEDRNAWRKTPLHTAAEKGHDSVALQLLEAGAKINATDHN  699
Burton’s mouthbrooder       VVDLLLSAKADPDATDNEKKTPLHLAAMEGKVDVAIS-LLSHRAKRRARDMDGSTPLHYAAAGGHVSVVTALLQPLNNKGTEDRNAWRKTPLHAAAEKGHDSVALRLLEAGAKINATDHN  700
Elephant shark              MADLLLSNHADANPLDKEKKTPLHLAASEGHLDFVSL-MLIRKGKFGAKDMDGNTPMLYAASKGHTSVVKAFLLAGRNHNVNDKNVWRRTALHLAAEHGSEDLVELLLMSGGAIDALDNA  677
Atlantic herring            VIKLLLSAGADVSATEKEKKTPLHMAAMEGKAEAVSSLLLQGKAKVEAKDMDGCTSLHYAAANGHIEAATALLSSGKIKSVDHKNTWRRTPLHAASEQGQEALVELLLSAGAKINATDSN  688
Zebrafish                   IIQSLLAAGAYSNASEREKKTPLHLAAAEGHTKAVSA-LLAGKAKVGAKDMDGCSPLHYAARNGKERAGSVLLASSKSKNVDDKNVWRRTALHLAAEHGHEALVGILLENKAKINALDNN  676
Blind cave fish             VIKLLLYAGADANAATKEKKTPLHLAATAGHAEATST-LLAGKANGAAKDMDGCSALHYAAGNGHESVAATLLTSGKNKNVDDRNVWRRTPLHLAAEHGQDILVDLLLEKQAKINAMDNN  681
African coelacanth          MVELLLNNNADPNSTDKEKKTSLHVAAAEGHSDVVEL-MLAKKAKFGAKDMDGCTPMHYASIQGNTEVVKALLAAGKNKNVDDKNVWRKTSLHLAAECGHNDMAELLLSSGASLNSLDNN  688
Western clawed frog         LVQLLLNNKADPNSTEKDKKTPLHIAAAAGHIEIVNV-MLKGRARCAVKDMDGCTPMHYAAATGSSEIAKALLKAGKNKNVDEKNVWRKTPLHLAAEHGHSDLINLLLQNGAAINALDNN  704
American chameleon          LVELLLGSNADPNSADKEKKTPLHLASIGSHLSTIKA-LLAKKSRFGAKDMDGCIPAHYAAISGNMEILKALLVAGNYKNINDKNIWRKTPLHLTAEYGHSDMMHYLLSNGSAINALDNN  674
King cobra                  MVELLLNFKADPNATDKEKKSPLHSATLGGHFYAVKA-LLAKKSRVASKDMDGCTPMHYAAISGNMEILKELLVAGDNKNINDKNIWRKTLLHLAAEHGQSNLIDFLLSNGSAINALDNN  682
Common garter snake         MVEMLLNFKADPNATDKEKKCPLHFATLGGHFYAVKA-LLAKKSKVASKDMDGCTPMHYAALSGNVEILKELLVTGNNKNINDKNIWRKTLLHLAAEHGHSNLIDFLLSNGSAINALDNN  682
Chinese alligator           MVELLINSNADVNVTDREKKAPLHMAAMGNYLNIVKL-LLAKKARFGAKDMDGCTPMHYASIKGNAEIVKLLLMEGKNKHVDDKNIWRKTPLHLAAEHGHSDLINLLLSHGSAINALDNN  686
Western painted turtle      MVELLLSFNADPNAMDKEKKTPLHTAAMGGHLNTVKV-LLAQKARFGIKDMDGCTPMHYATIKGNAEIVQILLTAGKNKNIDDKNIWRKTPLHLAAEHGHGDLINLLLSNGSAINALDNN  688
Green sea-turtle            MVELLLSFNADPNAMDKEKKTPLHTAAMGGHFNTVKV-LLAQKARIGIKDMDGCTPMHYATIKGNAEIVQILLTAAKNKNIDDKNIWRKTPLHLAAEYGHSDLINLLLSNGSTINALDNN  688
White-throated sparrow      LAEMLLKARADPNAQDKEKKTPLHAAALRGHLSIVEL-LLAKKGRAGAKDMDGCTPLHYATMKGNTDILKLLLASGKNKNINDRNVWRKTALHIAAEYGHGELINVLLSHGAAINASDSS  687
Chicken                     MVEMLLNANADPNAQDKEKKTPLHAAALRGHLSIVQV-LLAKKGRLGVKDMDGCTPMHYAAIKGNTEVVKLLLTSGKNKNIDDRNIWRKTALHIAAEYGHSDLINLLLGYGAAINALDSN  685
Turkey                      MVEMLLNANADPNAQDKEKKTPLHAAALRGHLGIVQV-LLAKKGRPGLKDMDGCTPVHYAAIKGNMEVEKILLTSTKSKNIDDRNIWRKTALHIAAEHGHSDLINLLLSYGAAINALDSN  685
Budgerigar                  VVEMLLNAKADPNAQDKEKKTPLHTAAVRGHLSIVEV-LLAGKARFGIKDMDGCTPMHYAAIKGNIEIVKILLASGKNKNIDDRNIWRKTALHIAAEHGHSDLINVLLSHGAAINASDSS  685
Golden eagle                MVEMLLNANADPNAQDKEKKTPLHTAAVRGHLSIVKV-LLANKGRHGAKDMDGCTPMHYAAVKGNIDIIKILLTSGKNKNIDDRNIWRKTALHIAAEYGHSDLINLLLSYGAAINALDSS  687
Barn owl                    MVEMLLNANADPNAQDKEKKTPLHIAAMRGHLSIVKA-LLAEKGRFRAKDMDGCTPMHYAAIKGNTEIVKLLLTLGKNKNIDDRNIWRKTALHIAAEYGHSDLINLLLSHGAAINALDSS  687
Spotted gar                 VVQLLLRNGADPNAVDKEKKSPLHMAALKGNSEAASA-MLSGKAMIRAKDMDGCTPLHYTTAKGHLSLMKVLLAFGKNKNVDDRNVWRKTALHIAAEHGYDSLVDFLLSSGAKINAMDIN  690
Asian bonytongue            VARLLLSTGADANAVDKEKKTPLHFAAMGGHGEVASV-LLARKASFRAKDMDGCTPLHYAAAKGHLDVAAVLLSTGK-KSVNDKNVWRKTALHLASEHGQDSLIDLLMCSGANVNALDNN  692
                            :   *:   :  .   .:**  ** *:           ::  :     :****     *::  *       :*     :  :.:* :*:* ** : * *   :   *:   . ::  *

Tasmanian devil             RDTPLHCACKTGHWSSVTSMINYSQG------EKPDLQAVNSLGKTPLQVAEGTVIENQEQIVTLLKKKMFLTR  757
Gray short-tailed opossum   RDTPLHCACKTGHWGSVTSLINCSQR------EKPDLQASNSLGKTPLQVAEGSATQSQEQIVTLLRKKMFLTR  750
Large yellow croaker        KDTPLHCAARGGHQEVVKRLVNWGHGGHMGRQKKANLQATNNVDKTPLQVAESGDTPQHEEIVTLLMRKMFLIK  774
Nile tilapia                KDTPLHCAARGGHHKVMKILVNWGQAGHVGWRNKANLQAKNNIGKTPLQVAESKHTPERENTAILLKRKMFLMK  773
Burton’s mouthbrooder       KDTPLHCAARGGHHKVMKILVNWGQAGNVGWRNKANLQAKNNIGKTPLQVAETKHTPEHENTAILLKRKMFLMK  774
Elephant shark              KDTPLHCACRSGNVGAAHRLANWAGG------QKAKFHVTNNVRKTPLQVSESGDSDGHRQIVVLLRKKMMLTR  745
Atlantic herring            KDTPLHCACRTGHLGIVQRLVNWAHK------EKANLQAINNVKKTPLQVAEAGDTLNHTHIVTLLKKKMFLMK  756
Zebrafish                   KDTPLHCACKTGHLGTVQRLINWTNG------ERANLQATNNVKKNALQVAEAGDTEAHENISTLLKKKMFLVK  744
Blind cave fish             KDTPLHSACRGGHVDTVQRLVNWTQG------QRANLHATNNVKKTAVQVAESEDSPNHQSIATLLKKKMFIIK  749
African coelacanth          RDTPLHSACRAGDLPTVQMLVNWTQG------KKGILQARNSRSKIPLEVAQSEGTHHHASIITFLKKKMLLIK  756
Western clawed frog         RDTPLHCACKAGHLSSVQTLVSWVQG------EKANLQATNGLKKTPLQVAESSSSDAHQHIATLLKKKMLIIR  772
American chameleon          KDTPLHCACKAGHFNCVNTLVNWSAG------EKVNLQATNSLKKTPLQTAESSSTDQQAQIVTLLKKKMLLIR  742
King cobra                  KDTPLHCACKAGHFDSVRALLNWSAG------DKANLQAANSLKKTPLQVAESSVTEHQAQIVNLLKKKMLLIR  750
Common garter snake         KDTPLHCACKAGHFDSVRALLNWSAG------DKANLQATNSLKKTPLQVAESSVTDQQAQIVNLLKKKMLLIK  750
Chinese alligator           KDTPLHCACKAGHFNSVNSLVSWSQG------ERANLQATNSLRKTPLQVAEFNTTENQAQIVTFLKKKMFITK  754
Western painted turtle      KDTPLHCACKAGHFNSVNSLVSWSQG------EKANLQATNSLKKTPLQVAEFSTSDNQGQIVTLLKKKMLIR-  755
Green sea-turtle            KDTPLHCACKAGHFNVVNSLVSWSQG------EKANLQATNSLKKTPLQVAEFSTSDNQAQIVTLLKKKKLVR-  755
White-throated sparrow      RDTALHCACRAGHLSAVSALLSWARG------EKANVLAVNSLRKTPLQVAEFHKTENQAQILTLLRKKMLIAK  755
Chicken                     KDTPLHCACKAGHLNAVNSLINWSQG------EKANLVAANSLKKTPLQVAECNKTENQAQIVTLLKKKMLMTK  753
Turkey                      KDTPLHCACKAGHLNAVNSLINWSQG------EKANLLAANSLKKTPLQVAECNKTENQAHIVNLLKKKMLMAK  753
Budgerigar                  RDTALHCACKAGHLNAVNALLNWSQG------QRANLLAANSLQKTPLQVAEFNITENQAQIVTLLKKKMLITK  753
Golden eagle                KDTPLHCACKAGHFNAANSLVNWSQG------EKANLLAANSLKKTPLQVAEFNKAENQAQIVKLLKKKMLITK  755
Barn owl                    KDTPLHCACRAGHFNAANSLVNWSQG------EKANLLAANSLKKTPLQVAEFNKTENQAQIVTLLKKKMLITK  755
Spotted gar                 KDTPLHIASRAGHLGTVHRLVNWTQG------EKVNLQATNSVKKTPLQVAQTGDTEKHQQVATLLKKKMFLIK  758
Asian bonytongue            KDTPLHCATRFGHLESVQRLLSWTGG------TGANLRAKNNVDKTPLQVAQCHHTANHQGIASLLQKKMLLIK  760
                            :** ** * : *.      : .              . . *   *  ::.::      :     :* :* ::
```

Figure S1. Sequence alignment of Caiap gene in different species. (A) Diagram showing the exons/introns organization of zebrafish caiap gene, indicating the position where the Caiap MO and gRNA bind. (B) Multiple alignment of zebrafish Caiap sequence with other identified Caiap sequences. The predicted CARD sequences are black boxed and yellow boxed indicate the ANK domains. (*) Identity in one position; (:) conservative substitutions in one position; (.) semiconservative substitutions in one position. The accession numbers for Caiap sequences are XP_685576 for zebrafish (*Danio rerio*), XP_007259539 for blind cave fish (*Astyanax fasciatus mexicanus*), XP_012694853 for atlantic herring (*Clupea harengus*), XP_006635081 for spotted gar (*Lepisosteus oculatus*), KPP59498 for Asian bonytongue (*Scleropages formosus*), KKF28470 for large yellow croaker (*Pseudosciaena crocea*), XP_006002757 for African coelacanth (*Latimeria chalumnae*), XP_005479286 for Nile tilapia (*Oreochromis niloticus*), XP_007901907 for elephant shark (*Callorhinchus milii*), XP_014189326 for Burton's mouthbrooder (*Haplochromis burtoni*), XP_008107583 for American chameleon (*Anolis carolinensis*), XP_013917140 for common garter snake (*Thamnophis sirtalis*), XP_006025802 for Chinese alligator (*Alligator sinensis*), XP_005284853 for western painted turtle (*Chrysemys picta bellii*), ETE70266 for king cobra (*Ophiophagus hannah*), XP_007059125 for green sea-turtle (*Chelonia mydas*), XP_010714589 for turkey (*Meleagris gallopavo*), XP_004936902 for chicken (*Gallus gallus*), XP_005151355 for budgerigar (*Melopsittacus undulatus*), XP_011570978 for golden eagle (*Aquila chrysaetos canadensis*), XP_009961493 for barn owl (*Tyto alba*), XP_005482422 for white-throated sparrow (*Zonotrichia albicollis*), XP_002931656 for western clawed frog (*Xenopus tropicalis*), XP_003767351 for Tasmanian devil (*Sarcophilus harrisii*), and XP_007480566 for gray short-tailed opossum (*Monodelphis domestica*).
