# Supplementary material for: Identification of an Evolutionarily Conserved Ankyrin Domain-Containing Protein, Caiap, Which Regulates Inflammasome-Dependent Resistance to Bacterial Infection
Source: Front Immunol. 2017 Oct 19;8:1375. doi: 10.3389/fimmu.2017.01375 (PMC5662874; doi:10.3389/fimmu.2017.01375)
Supplement: Supplementary file 4 [file image_2.pdf]

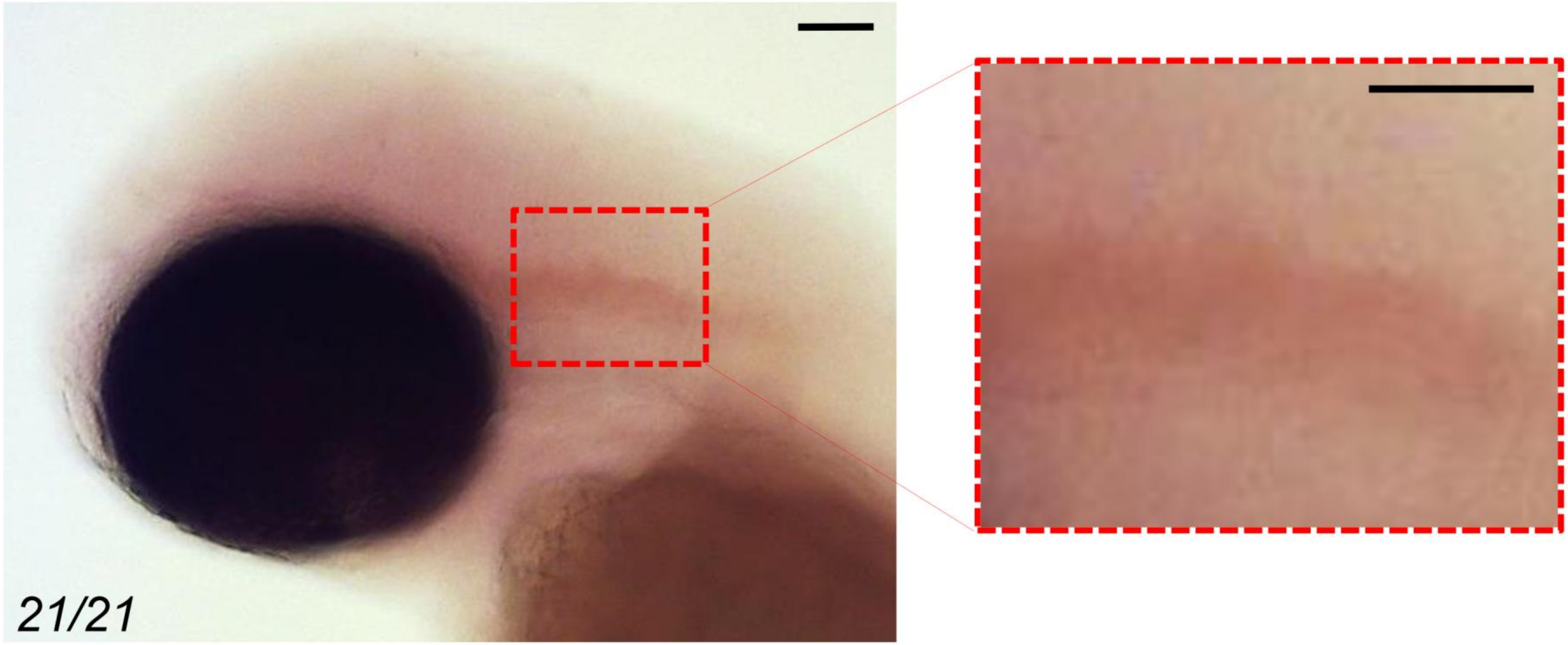

Figure S2. Control of WISH results shown in Figure 4. Zebrafish 2 dpf larvae were infected with ST in the otic vesicle. WISH was performed at 4 hpi using sense probes to the *caiap* gene. Note that no positive cells were observed at the infection site. The area shown is indicated in the larval scheme with boxes of different colors in Figure 4. Numbers in pictures represent the animals with the shown phenotype per total analyzed animals. Scale bar: 0.5 mm.
