# Supplementary material for: Identification of an Evolutionarily Conserved Ankyrin Domain-Containing Protein, Caiap, Which Regulates Inflammasome-Dependent Resistance to Bacterial Infection
Source: Front Immunol. 2017 Oct 19;8:1375. doi: 10.3389/fimmu.2017.01375 (PMC5662874; doi:10.3389/fimmu.2017.01375)
Supplement: Supplementary file 5 [file image_3.pdf]

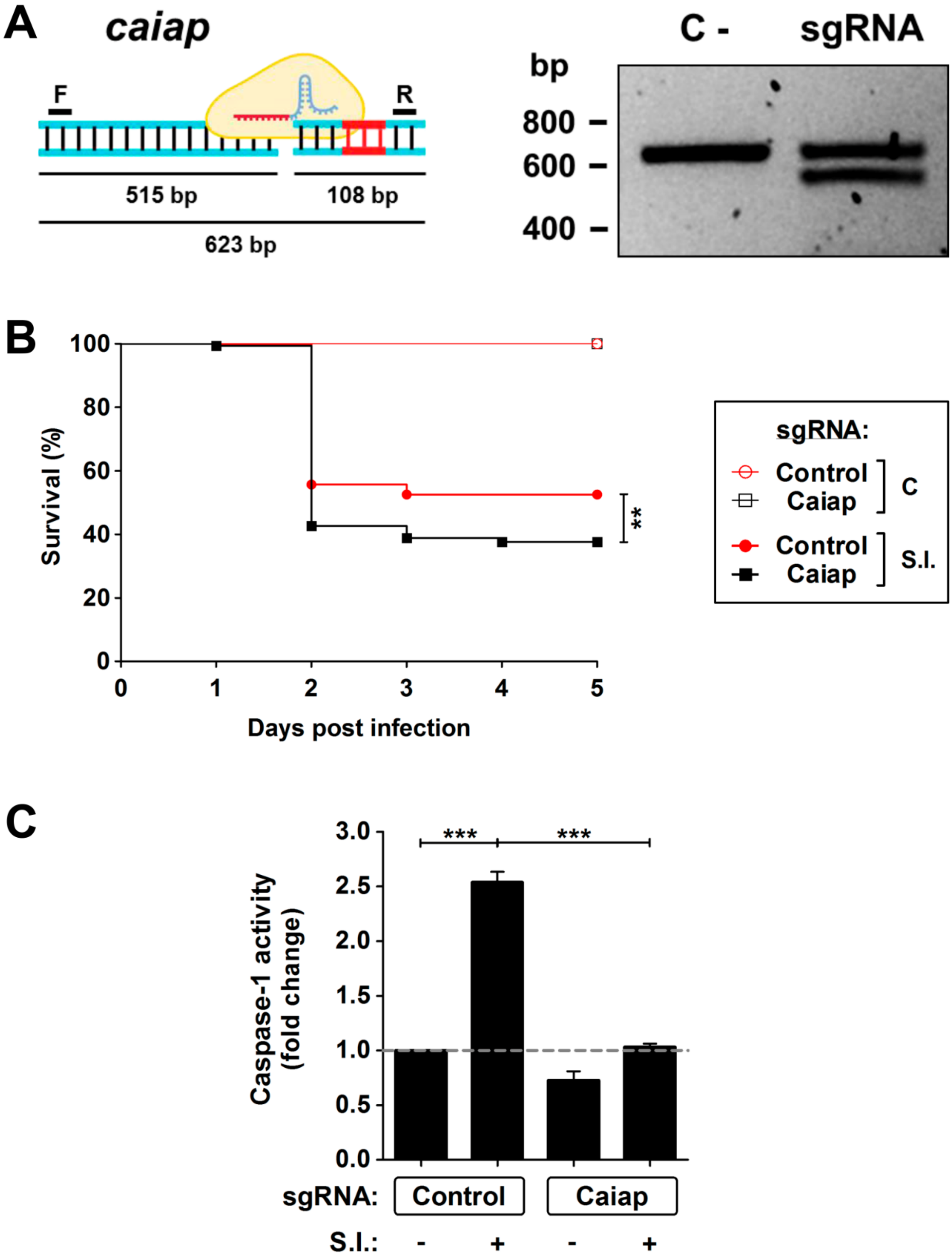

Figure S3. Caiap crispant larvae show high ST susceptibility and impaired caspase-1 activation. (A) Diagram showing the Caiap gRNA target at the 623 bp amplicon obtained with primers F and R, and the resulting digestion fragments. (B) Electrophoresis agarose gel stained with RedSafe showing the original amplicon in the sample incubated with Cas9 alone (C-) and the original amplicon and the expected 515 bp digestion fragment in the sample incubated with Caiap gRNA and recombinant Cas9. (C, D) Zebrafish one-cell embryos were injected with control or Caiap gRNA and recombinant Cas9. At 2 dpf, embryos were infected with ST and survival (C) and caspase-1 activity (D) were determined as described in Figures 5B and 5A, respectively. The sample size for each treatment is 300 for B and 30 for C. S.I., ST infection; **$p<0.01$; ***$p<0.001$.
